# Molecular and cellular characterizations of human cherubism: disease aggressiveness depends on osteoclast differentiation

**DOI:** 10.1186/s13023-018-0907-2

**Published:** 2018-09-20

**Authors:** Natacha Kadlub, Quentin Sessiecq, Marion Mandavit, Aurore Coulomb L’Hermine, Cecile Badoual, Louise Galmiche, Ariane Berdal, Vianney Descroix, Arnaud Picard, Amélie E. Coudert

**Affiliations:** 1 INSERM, UMRS 1138 Equipe 5, Laboratoire de Physiopathologie Orale Moléculaire, Centre de Recherche de Cordeliers, 75006 Paris, France; 2 Université Paris Descartes, 75006 Paris, France; 3 APHP, Necker Enfants Malades, service de Chirurgie Maxillo-faciale et Plastique, , Hôpital Necker Enfants Malades, 146 rue de Sèvres, 75015 Paris, France; 4 APHP, CRMR des Malformations Rares de la Face et de la Cavité Buccale, 75015 Paris, France; 5 CHU de Bordeaux, Service de Chirurgie Maxillofaciale, 33000 Bordeaux, France; 6 INSERM U 970, Equipe 10, PARCC, faculté Paris Descartes, 75015 Paris, France; 7 APHP, Hôpital Armand Trousseau, Service d’Anatomopathologie et cytologie, 75012 Paris, France; 8 Université Pierre et Marie Curie, 75005 Paris, France; 9 APHP, Hôpital Européen Georges Pompidou, Service d’Anatomopathologie et cytologie, 75015 Paris, France; 10 APHP, Necker Enfants Malades, Service d’Anatomopathologie et cytologie, 75015 Paris, France; 11 UFR Odontologie, Garancière, Université Paris Diderot, 75006 Paris, France; 12 APHP, Hôpital Pitié Salpétrière, Service d’Odontologie, 75013 Paris, France

**Keywords:** Cherubism, RANKL, TNF-α, Osteoclast, NFATc1, Auto-inflammatory bone disease

## Abstract

**Background:**

Cherubism is a rare autosomal dominant disorder of the jaws caused by mutation of the *SH3BP2* gene. The bone is replaced by a fibrous granuloma containing multinucleated giant cells. Cells of the cherubism granuloma have never been systematically analyzed. Hence, the aim of this study was to characterize the cells in human cherubism granulomas, to determine the osteoclastic characteristics of the multinucleated giant cells and to investigate the potential role of TNF-α in human cherubism.

**Results:**

Seven granulomas were analyzed in pathology, molecular biology and immunohistochemistry. Granulomas were composed mainly of macrophages or osteoclasts within a fibroblastic tissue, with few lymphoid cells. Myeloid differentiation and nuclear NFATc1 localization were both associated with disease aggressiveness. OPG and RANKL immunohistochemical expression was unexpected in our specimens. Five granuloma cells were cultured in standard and osteoclastogenic media. In culture, cherubism cells were able to differentiate into active osteoclasts, in both osteoclastogenic and standard media. IL-6 was the major cytokine present in the culture supernatants.

**Conclusion:**

Multinucleated giant cells from cherubism granulomas are CD68 positive cells, which differentiate into macrophages in non-aggressive cherubism and into osteoclasts in aggressive cherubism, stimulated by the NFATc1 pathway. This latter differentiation appears to involve a disturbed RANK-L/RANK/OPG pathway and be less TNF-α dependent than the cherubism mouse model.

**Electronic supplementary material:**

The online version of this article (10.1186/s13023-018-0907-2) contains supplementary material, which is available to authorized users.

## Background

Cherubism is a rare, autosomal dominant, benign fibro-osseous disease of the jaws with incomplete penetrance and variable expressivity [1]. The first signs appear between 2 and 5 years of age, progress until puberty, and then usually regress in adulthood [2]. Patients present a painless bilateral and multilocular granuloma of the jaws, affecting facial appearance and teeth eruption. Cherubism may also affect facial nerves, orbits and breathing [3, 4]. Histologically, cherubism granulomas are composed of a dense fibrous connective stroma with fibroblasts and randomly distributed multinucleated giant cells (MGC), considered to be osteoclast-like cells [5, 6].

At the molecular level, cherubism is caused by mutation of the *SH3BP2* gene (SH3 domain-binding protein 2), located on chromosome 4p16.3 [7]. SH3BP2 is an adaptor protein involved in lymphocyte activation, osteoclast differentiation and bone remodeling, through pathways involving Src, Syk and Vav-family protein kinases, and NFATc1 (nuclear factor of activated T cell 1) [8–13]. Most of the autosomal dominant mutations identified in cherubism lead to a single amino-acid change [7]. Recent genetic and biochemical studies have provided critical insights into the pathogenic mechanism of cherubism thanks to the creation of knock-in (KI) mouse models with the most common *SH3BP2* mutations [14]. However, unlike human *SH3BP2* heterozygotes, heterozygous mice do not exhibit any cherubism phenotype, and homozygous mutants develop severe bone loss due to osteoclast hyperactivity. Despite this important difference in genetic expression, *Sh3bp2* KI mice are considered a cherubism model [14]. According to Ueki’s mouse model, cherubism is associated with a high level of TNF-α (Tumor Necrosis Factor α) that is responsible for maintaining the phenotype: hyperactive macrophages secrete a high level of TNF-α that drives systemic inflammation, stimulates secretion of RANK-L (Receptor Activator of Nuclear factor Κ B Ligand) and M-CSF (Macrophage Colony Stimulating Factor) (osteoclastogenesis-associated proteins) by stromal cells, and ultimately results in bone loss [14]. In vitro, upon stimulation by RANK-L, *Sh3bp2* KI myeloid progenitor cells induce the activation of the NFATc1 signaling pathway, leading to hyperactive osteoclasts [14, 15]. In vivo, *Sh3bp2* KI mice develop systemic inflammation as a result of systemic infiltration by macrophages into tissues, as well as bone loss [14], defining cherubism as an auto-inflammatory bone disease [16–18].

The main objective of the present study was to determine if this auto-inflammatory bone disease paradigm could also be applied to human cherubism. To do so, we systematically examined the types of cells present in granulomas from 7 cherubism patients to look for evidence of chronic inflammation. We then characterized the osteoclastic features of the MGC both in vivo and in vitro. We also explored the potential role of TNF-α in the pathogenesis of human cherubism, and searched for potential biomarkers of the disease. Thus, we showed that in human cherubism, osteoclasts are the major myeloid cell type embedded within a fibrous stroma. The characteristics of these CD68-positive cells (macrophage vs. osteoclast) may predict the aggressiveness of the disease. Moreover, we demonstrated that first human cherubism granuloma is heterogeneous according to the patient and second the mechanism underlying human cherubism appeared to be different from that of mice.

## Methods

### Patients

This study included 7 patients (5 children and 2 adults) treated and followed in the maxillo-facial surgery department of Necker Hospital, Paris, through the MAFACE reference center for rare facial malformations. The 5 children were already part of our previously published cohort [19]. All patients gave their written informed consent for this study and for genetic analysis. Age, sex, age at diagnosis, age at first surgical treatment, radiologic extent and evolution of the lesions were recorded for each patient at the biopsy time. Medical treatment and past history were noted. Mutations in the gene encoding the binding protein SH3BP2 on chromosome 4p16.3 were sought for each patient. Direct Sanger sequencing of exons 2 to 13 of the *SH3BP2* gene was performed for 7 patients. All patients underwent intraosseous cherubism granuloma curettage.

The cherubism cases were classified according to patient age at the time of surgery (children were classified as group 1, adults as group 2) and sub-classified according to their aggressiveness based on the usual radiologic classification [20] and disease evolution after surgery [19]. For the child cases, radiologic grade I with favorable evolution after surgery was classified as group 1-A (low degree of aggressiveness: radiologic grade I with a favorable evolution); 1-B (moderate degree of aggressiveness: radiologic grade II-IV with a favorable evolution); 1-C (high degree of aggressiveness: radiologic grade V-VI and/or an unfavorable evolution, recurrence or extension); the adult patients were sub-classified as 2-A (remodeling bone) and 2-B (acute exacerbation) (Table 1) [20].

**Table 1:** Patient classification

Age	Classification	Definition	Patient	Age at surgery (years)	Gender	Radiological grade	*SH3BP2* mutation	Evolution
Child Patients	1-A	Low degree of aggressiveness: radiologic grade I with a favorable evolution after surgery	1-A	16	M	I.2	c.1244 G > A	Favorable
	1-B	Moderate degree of aggressiveness: radiologic grade II-IV with a favorable evolution after surgery	1-B1	9	F	II.1	c.1244 G > A	Favorable
			1-B2	8	M	II.1	c.1244 G > A	Favorable
	1-C	High degree of aggressiveness: radiologic grade I-VI with an unfavorable evolution after surgery	1-C1	8	M	V	c.1244 G > A	Unfavorable (recurrence and extension)
			1-C2	7	M	VI	c.1253 C > G	Unfavorable (recurrence and extension)
Adult Patients	2-A	Remodeling bone	2-A	19	F	N/A	c.1244 G > A	N/A
	2-B	Acute exacerbation	2-B	45	F	VI.3	c.1244 G > A	N/A

Cherubism cases were classified according to their age at surgery and sub-classified according to granuloma aggressiveness based on radiologic classification [20] and evolution after surgery. Age at the surgery, gender, radiologic grade, evolution and type of *SH3BP2* mutation are shown

### Sample collection

Granuloma samples were obtained at the time of surgery and divided into 2 parts, one for pathological examination and the second for the biochemical and biomolecular studies outlined below. Tissues for pathological examination were fixed in 10% formalin and embedded in paraffin. Four samples of normal alveolar bone, collected during third molar extraction, were included after written informed consent as controls for biomolecular analysis.

### HES staining

4-μm thick sections were cut from each paraffin block. Section staining with hematoxylin/eosin/safranin (HES) was automated by using a Leica Autostainer (Nussloch, Germany): after deparaffinization (in successive baths of xylene and alcohol), staining was performed by successive baths in hematoxylin GILL2 (Thermo Shandon, Pittsburgh, PA, USA), 1% eosin (Ral Diagnostics, Martillac, France), 0.12% safranin in 1% hydrochloric acid solution (Ral Diagnostics, Martillac, France) and 95% ethanol. The sections were then mounted in synthetic resin (WVR, Radnor, PA, USA). Two pathologists blinded to clinical history scored the slides, according to the previous description of cherubism [5, 21, 22]. For each specimen, we evaluated presence of intracytoplasmic vacuoles in MGC, presence of collagen (semi quantitative evaluation: 0, +, ++, +++), cells subtype (fibroblast, round cells, MGCs) semi-quantitative evaluation (0, +, ++, +++), perivascular hyalinosis. From 5 randomly selected areas at 200 high-power field (HPF), we evaluated the number of MGC and the number of nuclei per MGC.

### Immunohistochemistry

#### Vimentin*,* AE1-AE3, CD68, CD4, CD8, CD3, CD5, CD20 automated immunohistochemistry (Additional file 1)

Immunohistochemical staining was performed using a Ventana Benchmark® XT automated slide preparation system (XTUltraviewDABv3, Ventana, Tucson, AZ, USA). 4-μm thick sections were incubated with primary antibody (Table 2). After washing in phosphate-buffered saline (PBS), sections were incubated with streptavidin/horseradish peroxidase (Biolegend, San Diego, CA, USA). Sections were immersed in 3% H_2_O_2_ to quench endogenous peroxidase activity. Staining was visualized in brown color using a diaminobenzidine tetrahydrochloride chromogen substrate (DAB, SK-4105 Vector Laboratories, Burlingame, CA, USA). Sections were counterstained with hematoxylin. Slides were scored by two pathologists blinded to clinical history and the primary antibody used. The number of positive cells was evaluated for each assay from five randomly selected areas (200 HPF), except for vimentin. Vimentin expression was semi-quantitatively evaluated (0 = vimentin negative; + few positive cells; ++ less than the half positive cells; +++ more than half positive cells).

**Table 2:** RANK/RANKL/OPG triad and NFATc1 expression by cherubism-granuloma cells

	RANK		RANK-L		OPG		NFAT-c1	
	Stromal cells (cytoplasmic)	CMGC (cytoplasmic)	Stromal cells	MGC	Stromal cells	MGC	cMGC (cytoplasmic)	cMGC (Nuclear)
1-A	–	–	–	–	–	–	–	–
1-B_1_	+	+	–	–	–	–	+	–
1-B_2_	–	–	+	–	–	–	–	–
1-C_1_	+	+	+	–	+	–	+	+
1-C_2_	+	+	+	–	+	–	+	+
2-A	–	–	–	NA	+	–	–	–
2-B	–	–	+	–	–	–	+	+

Expression of each protein was evaluated for MGC and stromal cells. For NFATc1, numbers of nuclear and cytoplasmic positive cells were evaluated. For each assay five randomly selected areas were evaluated (200× high-power field)

#### RANKL, RANK (receptor of activated nuclear factor Κ B), OPG (osteoprotegerin), TNFR1(tumor necrosis factor receptor 1)., IL6 (interleukin), IL17, NFATc1 manual immunohistochemistry (Additional file 2)

For immunohistochemistry staining, 4-μm thick sections were first deparaffinized with xylene for 30 min, post-fixed with 90% ethanol for 10 min and then washed in distilled water for 5 min. For antigen retrieval, the sections were incubated in a 78 °C water bath for 30 min in pH 6 or pH 9 buffer, cooled at room temperature for 20 min, washed in PBS for 10 min and finally incubated with reagents from an avidin/biotin kit (Vector Laboratories, Perterborough, UK). Endogenous perodixase activity was blocked by incubating the sections with 3% H_2_O_2_ followed by a wash in PBS. Sections were blocked in a 5% normal human serum for 30 min before incubation with the primary antibodies or isotype control for 60 min at room temperature. After a PBS wash, the sections were then incubated with the secondary antibody for 30 min, then with streptavidin/horseradish peroxidase for 30 min (Biolegend, San Diego, CA, USA). Staining was visualized as a brown color by using a diaminobenzidine tetrahydrochloride chromogen substrate (Vector Laboratories, Perterborough, UK). Slides were scored by two pathologists blinded to clinical history and the primary antibody used. The signal for each antibody was evaluated for MGCs and stromal cells. For NFATc1, the numbers of nuclear and cytoplasmic positive cells were evaluated.

### TRAP (tartrate resistant acid phosphatase) activity assay

A TRAP activity assay was performed on paraffin-embedded tissue sections and cell cultures from granulomas on Lab-Tek™ chamber slides (Dutscher). Paraffin sections were deparaffinized and rehydrated, stained in pH 5.2 acetate buffer containing 2.5 mM Naphthol AS-TR phosphate, 0.36 M N–N dimethylformamide, 0.1 M sodium tartrate and 4 mM Fast Red TR Salt (Sigma-Aldrich). Hematoxylin was used for nuclear staining. TRAP-positive giant multinucleated (> 3 nuclei) cells were considered as osteoclasts. The number of osteoclasts (MGC > 3 nuclei) and number of nuclei per osteoclast were evaluated from five randomly selected areas (200 HPF).

### RNA extraction, reverse transcription and qPCR

Total RNA was extracted from the granuloma samples, bone controls or cell cultures using Trizol Reagent (Life technology, Saint Aubin, France) according to the manufacturer’s instructions. The total RNA yield (ng) was determined fluorometrically using a Qubit fluorometer (Life Technologies, Saint Aubin, France). Total RNA (1 μg) was reverse transcribed using SuperScript II™ reverse transcriptase (Life Technologies, Saint Aubin, France) according to the manufacturer’s instructions. Real-time quantitative PCR was carried out using SYBR-green master mix (Life Technologies, Saint Aubin, France) in a BCR Bio-Rad Opticon thermocycler (Bio-Rad, Marne La Coquette, France). PCR conditions were: 98 °C for 30 s followed by 40 cycles of 95 °C for 10 s, 60 °C for 20 s and 72 °C for 20 s. Primer sequences of all the analyzed genes are shown in (Additional file 3). Cq was transformed into quantity values using the formula (1 + Efficiency)^-Cq^ as previously described [23]. *GAPDH*, *SDHA*, *TBP* and *HPRT* were used as reference genes.

### Granuloma cell culture

Samples from only 5 cherubism cases yielded cell cultures. Tumor tissues were washed in PBS. Fragments of the tumors were incubated at 37 °C for 60 min in a hyaluronidase and collagenase solution mix (1 mg/ml for each enzyme) in PBS. The number of cells was counted manually using a Mallassez counting chamber. Cells were cultured either on a dentin slice (ids, Paris, France) (at a density of 0.5 × 10^6^ cells/ml) for the resorption assay, Lab-Tek II chamber slides 1 well (ThermoScientific, Rochester, NY, USA) (at 0,6 × 10^6^ cells/cm^2^) for the ELISA supernatant study, or Lab-Tek II chamber slides 8 wells (at 0,6 × 10^6^ cells/cm^2^) for the TRAP activity assay with standard medium. After 12 h of incubation at 37 °C in a 5% CO_2_ humidified atmosphere, half of the cell culture was incubated in standard medium and the other half in osteoclastogenic medium. Standard medium contained minimal essential medium (αMEM, without red phenol) and 1% Penicillin-streptomycin, 1% L-glutamine (all from Life Technologies, Saint Aubin, France) and 10% fetal calf serum (Hyclone, South Logan, UT, USA). For the Lab-Tek cultures, osteoclastogenic medium contained standard medium supplemented with RANKL at 30 ng/ml and M-CSF at 25 ng/ml (Peprotech, Neuilly-sur-Seine, France). For the dentin slices cultures, osteoclastogenic medium contained standard medium supplemented with RANKL at 60 ng/ml and M-CSF at 25 ng/ml (Peprotech, Neuilly-sur-Seine, France). Half of the cell culture was maintained for 3 days, and the other half was maintained for 7 days. In the 7-day cultures, the culture medium was changed at day 3. Supernatants were collected at day 3 and day 7 for ELISA. Cultures in Lab-Tek 8 chambers were fixed in 4% paraformaldehyde for TRAP activity measurement, and cultures in Lab-Tek 1 chambers were treated with Trizol Reagent for RNA extraction. Cultures on dentin slices were washed in distilled water and sonicated to remove cells. The slides were then stained with 0.5% toluidine blue to reveal lacunar resorption areas by light microscopy.

### Osteoclast in vitro differentiation from peripheral blood mononuclear cells (PBMC)

Whole blood (15 ml) was obtained from the French Transfusion Establishment (EFS). Blood was diluted with 1X PBS, layered on 12 ml of Ficoll (Euromedex, Souffersheim, France), and then centrifuged (× 1500 g, 10 min, room temperature, with the brake off). The PBMC layer was collected and washed in PBS, and then counted in a Malassez counting chamber. The cells were plated in Lab-Tek 8 chambers (at 0,6 × 10^6^ cells/cm^2^), in standard medium and osteoclastogenic medium for 14 days.

### ELISA

Levels of RANK-L, OPG, M-CSF, TNF-α and IL-6 in culture supernatants were determined by commercially available specific ELISAs, according to the manufacturer’s protocols (R&D Systems, Minneapolis, MN, USA). The lower and higher detection limits for each cytokine are shown in Additional file 1. It is important to note that the system only detects free, unbound RANK-L. Absorption was determined with an ELISA reader at 450 nm (Additional file 4).

### Statistical analysis

Results are expressed as mean ± 2 SEM. Statistical comparisons were made using ANOVA, with *p* < 0.05 being considered significant.

## Results

### Patients

Seven patients with cherubism, five children and two adults, were investigated in this study. Patient ages ranged from 6 to 45 years old at the time of surgery (intraosseous granuloma curettage). None of the patient presented intercurrent disease or medical therapy. The children were categorized as group 1 and the adults as group 2. They were further classified according to granuloma radiological grade and evolution after surgery (Table 1, see Materials and Methods for details). Six of the patients had the same *SH3BP2* mutation (c.1244 G > A; p.Arg415Gln), and one had the most common *SH3BP2* mutation (c.1253 C > G; p.Pro418Arg), previously described ^7^. Noteworthy was the wide variation in cherubism aggressiveness in this patient sample.

### Cherubism biomarker search

To identify a potential cherubism biomarker, RNAs from a granuloma sample from each patient were extracted to establish a molecular profile for each patient’s granuloma. As cherubism was recently described as being an auto-inflammatory bone disease, we focused on the expression of genes involved in osteoclastogenesis (RANK, RANKL, M-CSF, OPG, NFATc1), bone formation (ALP, Osteocalcin), and inflammation (IL6, IL6R, TNFα, TNFR1, TNFR2, IL17) (Additional file 5).

All granulomas (except 1-B1) and the bone controls provided suitable material for RNA extraction and RT-qPCR analysis. The granulomas and bone expressed M-CSF, TNF-α, TNF-R1, and TNF-R2 mRNAs with no significant differences between the granuloma and bone. RANK mRNA was expressed by all samples, and significantly more expressed in samples 1-B2 (*p* = 0.07) and 1-C1 (*p* = 0.0003). RANK-L mRNA was expressed by all samples, and significantly (*p* = 0.0121) more expressed in sample 1-B2. OPG mRNA was expressed by all samples, and significantly (*p* = 0.002) more expressed in sample 2-A. The RANKL/OPG ratio was positive in all cases except sample 2-A. NFATc1 mRNA was expressed by all samples, and significantly (*p* = 0.0001) increased in 1-C2. Osteocalcin and ALP mRNA were expressed by all samples, and significantly more expressed in 2-A (*p* = 0.00001). IL6-R mRNA was expressed by control bone, 1-C1 and 2-B, with a significant increase in 1-C1 (*p* = 0.0287). Granulomas and control bone did not express IL-6 and IL17 mRNAs.

**Fig. 1:**
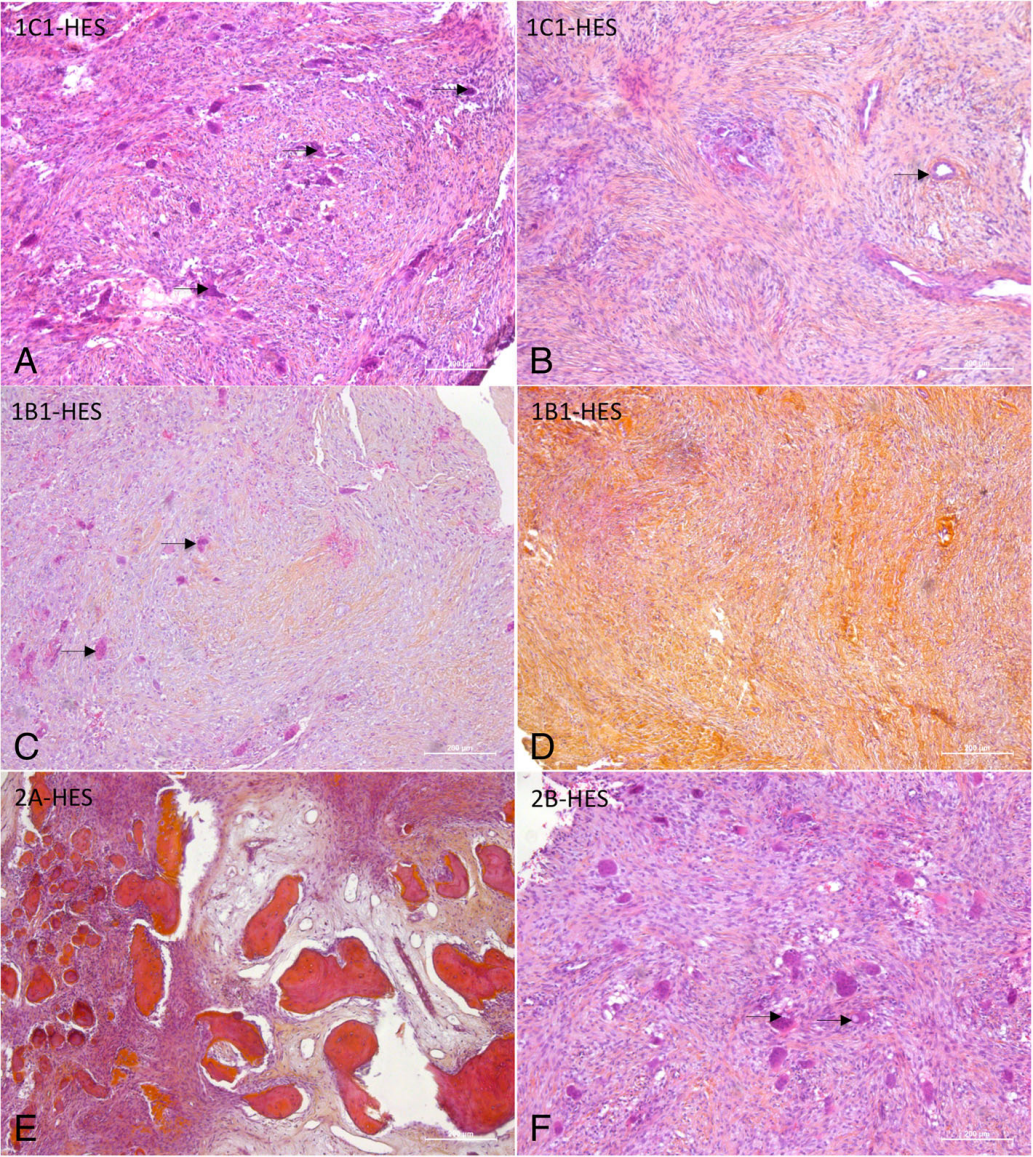
Histopathological characteristics of cherubism granulomas. Granuloma sections were stained with HES (hematoxylin-eosin-safranin). Cherubism granulomas are heterogeneous lesions both within a granuloma and among patients (scale bar 200 μm). **a** Case 1-C1 showed multinucleated giant cells (black arrows) (MGC), mononuclear cells and collagen (safaranin-orange coloration) stroma. **b** Case 1-C1 showed fibrous collagen-rich stroma, vessels (black arrow) with hyalinosis cuffing. **c** Case 1-B1 showed MGC (black arrows), with mononuclear cells and a collagen (safranin)-rich stroma. **d** Case 1-B1 showed a fibrous collagen-rich stroma. **e** Case 2-A showed immature bone within a fibrous stroma. **f** Case 2-B showed MGC with intracytoplasmic vacuoles (black arrows), mononuclear cells and collagen (safaranin-orange coloration) stroma

Biomolecular analysis showed that, in adult patient 2-A, in whom bone remodeling had occurred post-surgery, the biomolecular profile indicated bone synthesis: increased expression of osteoblast markers (ALP, osteocalcin), and a RANK-L/OPG ratio greater than 1. However, the overall results of the biomolecular analysis reflected extensive inter-tumor heterogeneity (Fig. 1a-d) and did not allow us to define specific cherubism biomarkers.

### Identification of the cell types present in cherubism granuloma (Additional files 6, 7 and 8)

We first began with a simple histologic analysis. Hematoxylin-eosin-safranin-stained sections of six of the cherubism granulomas (excluding the remodeling case, 2-A) showed multinucleated giant cells within a collagen-rich and well-vascularized fibrous stroma (Fig. 1a-d). The granuloma histology was heterogeneous between patients, reflecting their different cherubism grades, and within each granuloma (Fig. 1a-d). Hyalinosis cuffing surrounded all vessels (Fig. 1b, d). The number of MGC varied from 0 to 35 cells per HPF (mean 6.2 ± 2.1). MGC had 3 to 23 nuclei per cell (mean 7.17 ± 1.25), and some MGC presented intracytoplasmic vacuoles (3 of 7 granulomas). Collagen was abundant (++, +++) in poor-cell- area, and rare (0, +) in rich-cell area (Fig. 1a, d). Reactive bone was seen in all tumors. Adult case 2-A (the remodeling one) showed immature bone in a fibrous stroma, without MGC (Fig. 1e). The acute exacerbation in adult case 2-B showed MGC within a collagen-rich and well-vascularized fibrous stroma, as in the child cases (Fig. 1f).

**Fig. 2:**
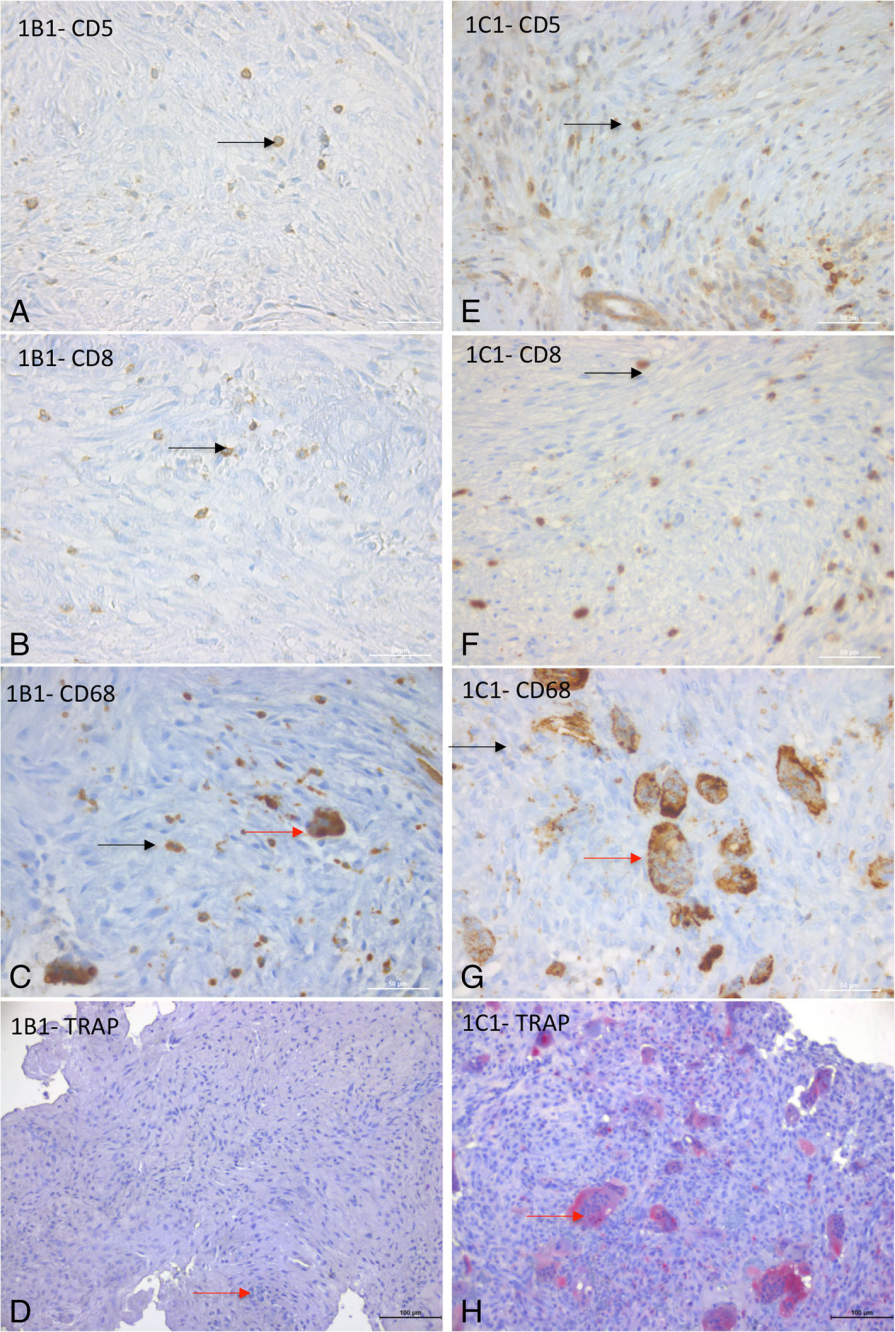
Characterization of cells present in cherubism granulomas (CD5, CD8, CD68, TRAP activity). (CD5, CD8, CD68 scale bar = 100 μm; TRAP activity scale bar = 50 μm). **a**-**d**: Case 1-B1, child with moderately aggressive cherubism: granuloma is composed of fibroblastic stroma with CD8+ cells and monocytes. **a** Identification of lymphoid cells (black arrow: CD5-positive cells) within the stroma. **b** Identification CD8+ lymphoid cells (black arrow: CD8-positive cells) within the stroma. **c** Identification CD68-positive cells, principally composed of monocytes (black arrow: mononuclear CD68-positive cell); and few macrophages (red arrow: multinuclear CD68-positive cell). **d** TRAP assay (× 20) showed that MGC are TRAP negative (considered as macrophages: red arrow)*.* **e**-**h** Case 1-C1_,_ child with highly aggressive cherubism. Granuloma is composed of fibroblastic stroma with CD8+ cells and osteoclast-like cells. **e** CD5 staining showed lymphoid cells within the stroma (black arrow). **f** CD8 staining showed CD8+ lymphoid cells within the stroma (black arrow). **g** CD68 staining showed cells principally composed of macrophages (multinuclear CD68-positive cell, red arrow) and monocytes (mononuclear CD68-positive cell, black arrow). **h** TRAP assay showed numerous TRAP-positive MGC (osteoclasts, red arrow). TRAP: Tartrate Resistant Acid Phosphatase

Because, based on a mouse model, cherubism is considered to be an auto-inflammatory disease [16, 17], we characterized the immune cells to look for an indication of chronic inflammation. Some mononuclear cells were lymphocytes, CD5^+^. Among these lymphocytes, most were CD8+ T lymphocytes, some were CD4^+^ cells, and fewer still were CD20^+^ cells (Table 3, Fig. 2a, b, e, f). In addition, our analysis showed that the cells that expressed CD68^+^ (antigen expressed by myeloid cells) could be mononucleated or multinucleated (Table 3, Fig. 2c, g). In addition, all MGC expressed CD68.

**Table 3:** Identification of cherubism granuloma cells (CD5, CD3, CD4, CD8, CD20, CD68, AE1/AE3, Vimentin, TRAP activity)

	Cells/20HPF									
	CD5 Lymphocytes	CD20 B-Cells	CD3 T-Cells	CD4 LT4	CD8 CD8+	CD68 mononuclear Monocytes	CD68 Multinuclear Osteoclast-like	Vimentin Semi-quantitative	AE1/AE3	TRAP Osteoclast-like
1-A	15 [9–20]	0	14.8 [8–18]	0	14.6 [8–21]	5.4 [3–9]	1.4 [0–3]	+++	0	0
1-B1	8 [6–9]	0.2 [0–1]	7 [4–9]	0.6 [0–2]	6.8 [2–9]	18 [9–32]	3.2 [1–5]	+++	0	4 [0–6]
1-B2	2.6 [0–7]	0	1.6 [0–3]	0.6 [0–1]	2 [1–3]	34.24 [31–37]	2.8 [1–5]	+++	0	0
1-C1	19.2 [8–37]	2.4 [0–6]	29.2 [18–30]	4.4 [0–11]	21.6 [6–32]	14 [6–22]	8,2[0–10]	+++	0	8.6 [8–10]
1-C2	31 [15–48]	0.2 [0–1]	19.6 [15–27]	4.2 [0–10]	17 [4–37)	21.8 [10–35]	13.2 [7–20]	+++	0	7.4 [3–11]
2-A	0.6 [0–2]	0	0	0	0	0.8 [0–2]	0.4 [0–2]	+++	0	0
2-B	21.4 [10–33]	0.6 [0–2]	31.6 [27–35]	0.6 [0–2]	7.2 [3–15]	14.6 [8–19]	2.8 [2–6]	+++	0	3.2 [1–5]

Number of positive cells was evaluated for each assay from five randomly selected areas (200× high-power field; HPF). Vimentin expression was semi-quantitatively evaluated (0 = vimentin negative; + few positive cells; ++ less than the half positive cells; +++ more than half positive cells). CD5 identifies lymphoid cells; CD3, T cells; CD4, T4 cells; CD8, CD8 + cells; CD20, B cells; CD68 determines monocyte lineage cells, immature macrophages (mononucleated cells), macrophages or osteoclasts (multinucleated cells); AE1/AE3 identifies epithelial cells, vimentin mesenchymal cells, TRAP activity in osteoclast cells when multinucleated (> 3 nuclei)

**Fig. 3:**
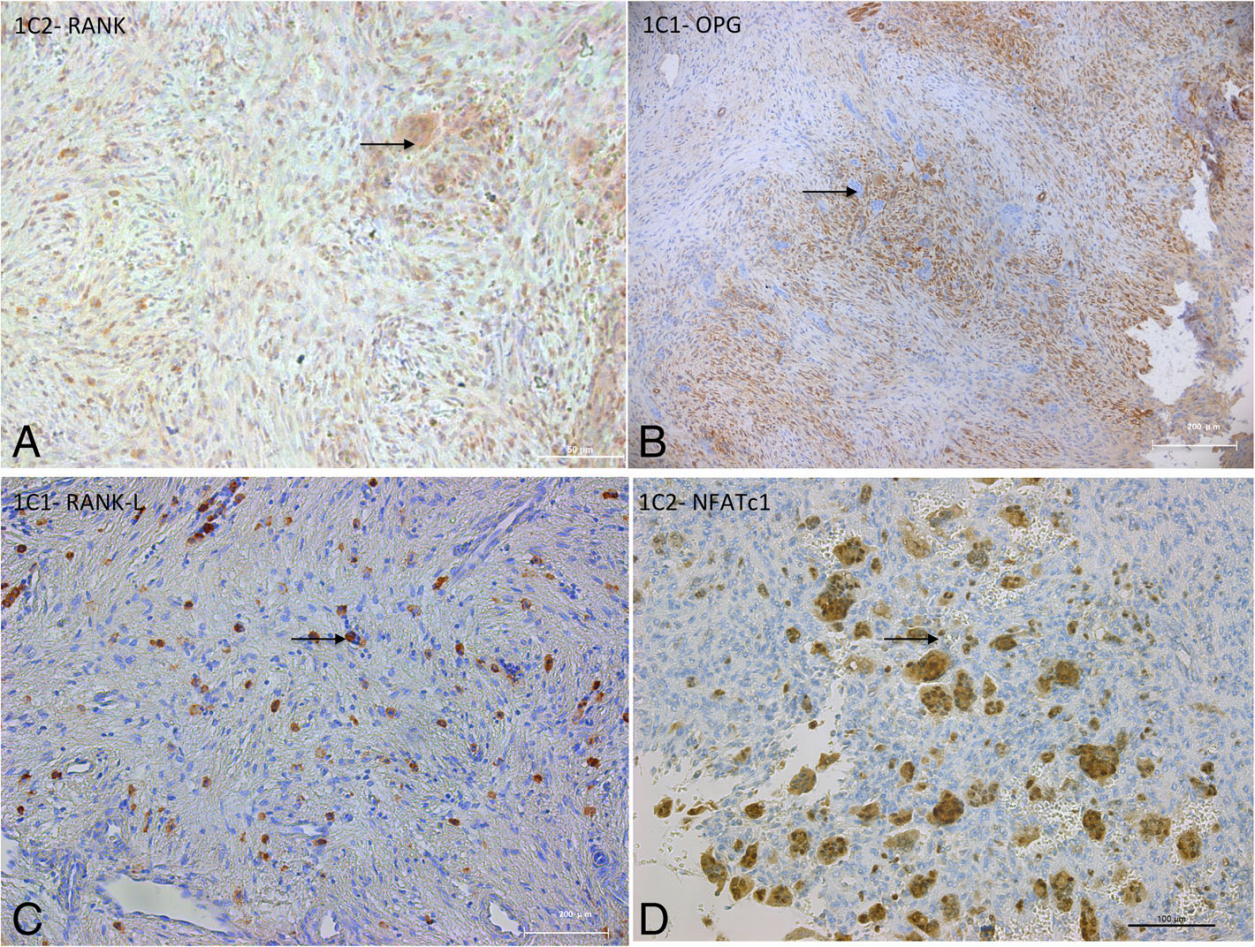
Bone remodeling marker: RANK-L, OPG, RANK, NFATc1 immunohistochemistry. **a** Case 1-C2 showed that both stromal and MGC cells express RANK in their cytoplasm (black arrow) (scale bar = 50 μm). **b** Case 1-C1 showed that stromal cells around MGC (black arrow) express OPG (scale bar = 200 μm). **c** Case 1-C1 mononuclear stromal cells (black arrow) distant from MGC express RANKL (scale bar = 200 μm). **d** Case 1-C2 showed expression of NFATc1 in MGC nuclei and cytoplasm (scale bar = 100 μm)

To determine if CD68^+^ multinucleated cells were osteoclasts, we performed a TRAP activity assay. Some MGC CD68^+^ cells were positive for TRAP. The aggressive tumor of patient 1-C showed a high number of TRAP-positive MGC (considered as osteoclast-like cells when TRAP positive with more than 3 nuclei) (Fig. 2d, h, Table 3). Then, to address the role of the RANK/RANKL/OPG triad in cherubism and in the development of these MGC osteoclast-like cells, we studied the expression of these proteins essential for osteoclastogenesis [24]. RANK was expressed in both stromal cells and MGC (Fig. 3a). We found that stromal cells expressed osteoprotegerin (OPG) near the MGC (Fig. 3b). In areas without MGC, some stromal cells of granulomas 1-B2 and 1-C1 expressed RANK-L (Fig. 3c). In the most aggressive cherubism, cases 1-C1 and 1-C2, and in acute adult cherubism, case 2-B, MGC expressed both nuclear and cytoplasmic NFATc1 (Fig. 3d) (Table 2).

Other cells present were supposed to be fibroblasts as they were CD5^−^, CD68^−^, AE1/AE3^−^, vimentin +, and spindle shaped (Fig. 2, Table 3).

Finally, because inflammatory cytokines have been implicated in cherubism [14, 25], we analyzed cytokine receptor expression on the granulomas. No expression of TNF-R1–2 was observed. A few stromal cells from cases 1-A, 1-C1, and 2-B expressed IL6-R (data not shown).

Overall, the histopathological analysis revealed great heterogeneity of cherubism granulomas, in part due to the aggressiveness of the disease; and the unexpected and atypical expression of the RANK/RANKL/OPG triad in cherubism granulomas (low expression of RANKL by cells outside the MGC area; high expression of OPG around the MGC; variable expression of RANK by both MGC and stromal cells) (Table 2). Consistent with an auto-inflammatory bone disease [16, 17], lymphoid cells were present (CD5^+^), most of which were CD8+ lymphocytes; however, immature macrophages (CD68^+^ mononuclear cells), mature macrophages (CD68^+^, multinucleate, TRAP-) and osteoclasts (TRAP+, multinucleate with > 3 nuclei) were observed. TRAP activity, NFATc1 staining and the number of nuclei in CD68^+^ cells, strongly suggesting an osteoclastic phenotype, seem to be associated with the aggressiveness of cherubism. In non-aggressive cherubism, CD68^+^ cells displayed a macrophage phenotype, whereas in aggressive cases, CD68^+^ cells displayed osteoclast characteristics (> 3 nuclei, TRAP+, nuclear NFATc1+).

### Osteoclastic phenotype of multinucleated giant cells from cherubism granulomas in vitro

**Fig. 4:**
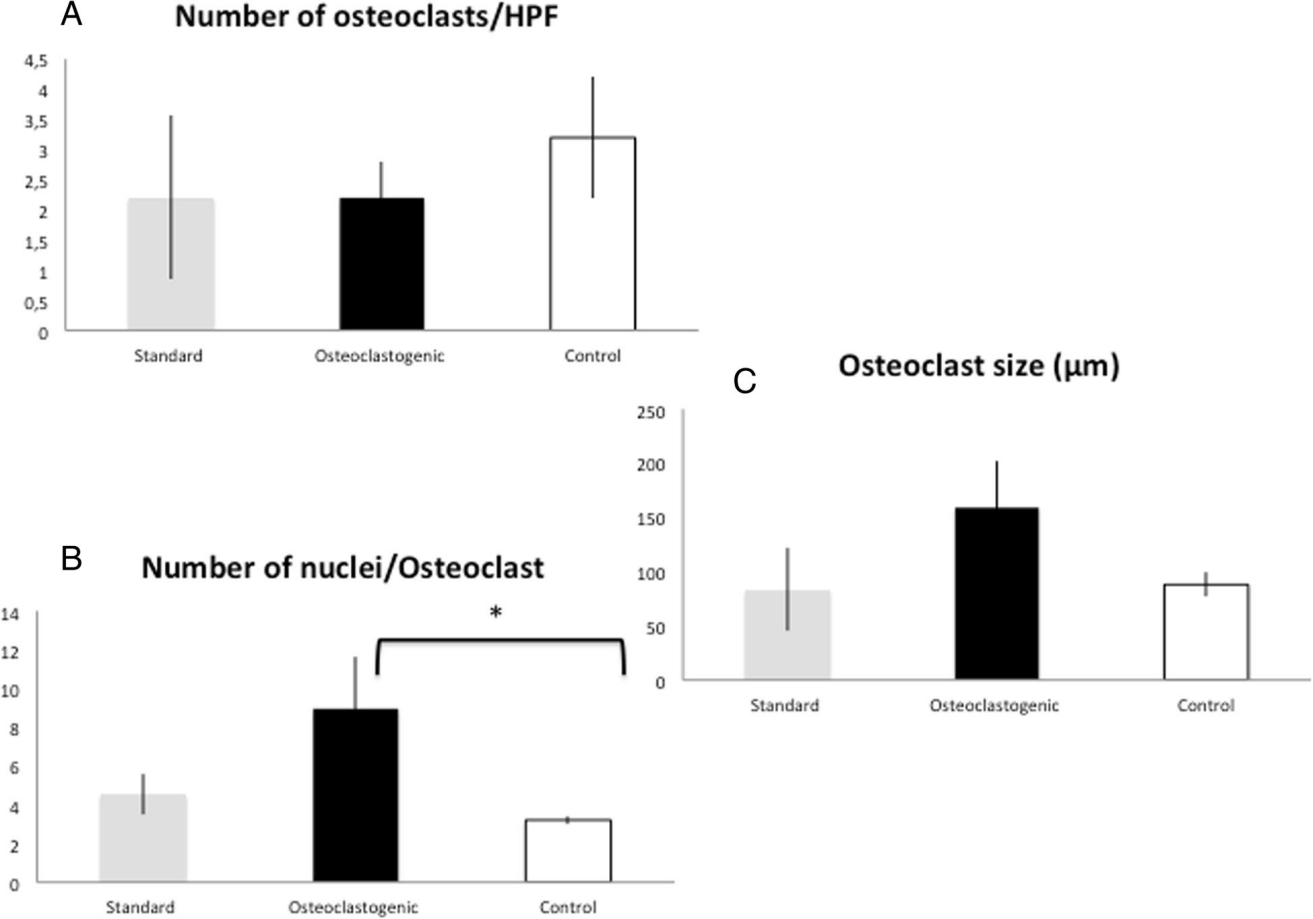
Cherubism giant multinucleated cells differentiate into osteoclasts in culture. Cultures in standard and osteoclastogenic media of cherubism granuloma at day 7 (cases 1B-1, 1B-2, 1-C1, 1-C2, 2-B) compared to osteoclasts obtained from healthy-donor PBMC-differentiation cultures at day 14 (control). 1-A: Cherubism differentiates into osteoclast (> 3 nuclei, TRAP^+^ cells) at day 7 in standard and osteoclastogenic media. Number of osteoclasts at day 7 in cherubism is not significantly different from the PBMC culture at day 14. 1-B: Number of nuclei of cherubism osteoclasts at day 7 cultivated in osteoclastogenic medium is significantly increased compared to the number of nuclei in osteoclasts from PBMC culture at day 14 (*p* = 0.002). 1-C: Size of cherubism osteoclasts at day 7 cultivated in osteoclastogenic or standard medium is not significantly different from the size of healthy donor osteoclasts

**Fig. 5:**
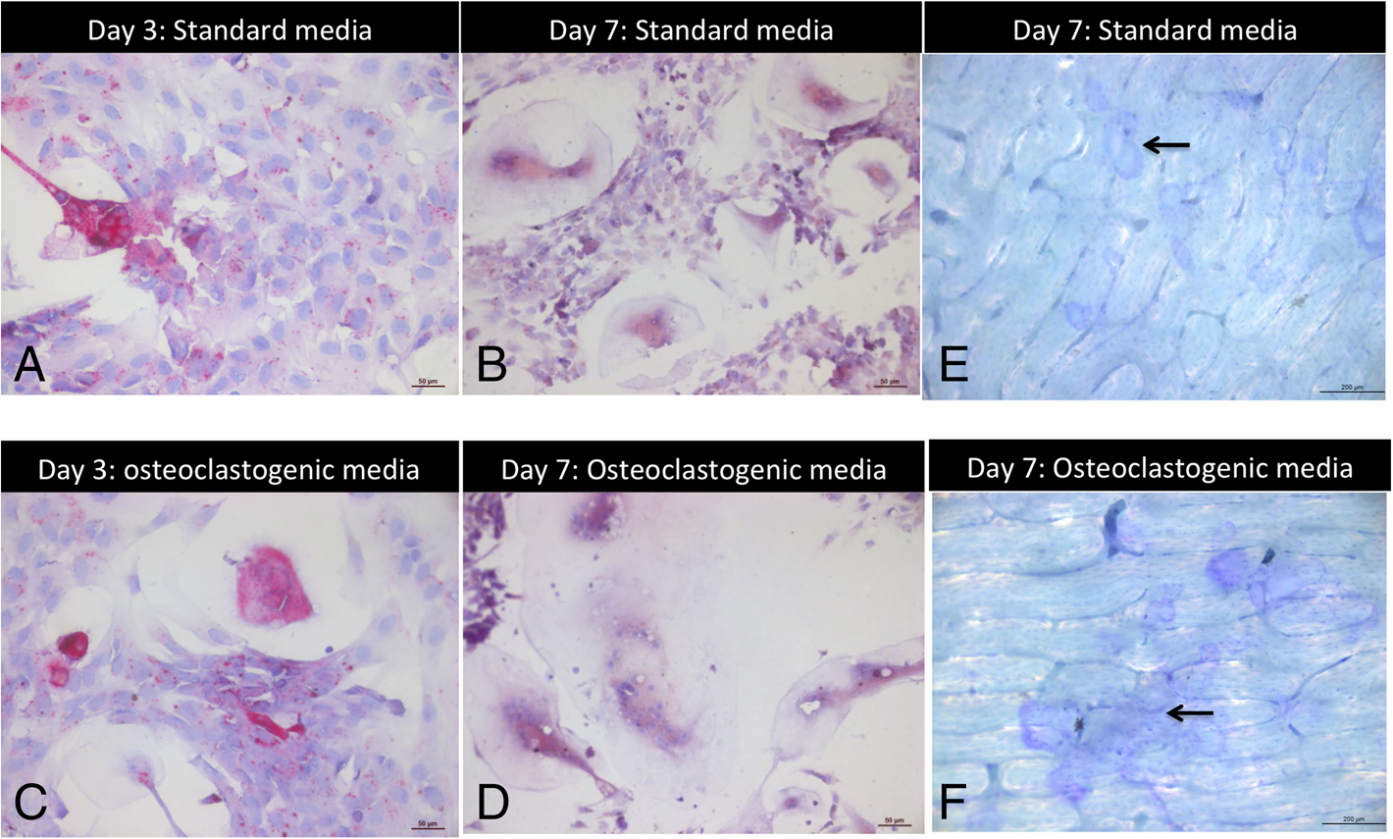
Cherubism MGC are osteoclasts. **a**-**d**: TRAP Assay, × 20 (scale bar = 50 μm), E and F: Resorption assay × 10 (scale bar = 200 μm). **a**-**c**, Case 2-B. Cherubism cells cultured in standard (**a**) and osteoclastogenic (**c**) medium at day 3 showed that MGC are TRAP-positive, **b**-**d** Case 2-B. Cherubism cells cultured in standard (**b**) and osteoclastogenic (**d**) medium at day 7. Osteoclast number, osteoclast size and nuclei number increased from day 3 to day 7. **e**-**f** Case 2-B. Bone slices displayed pit formation at day 7 in standard (**e**) osteoclastogenic medium (**f**) (arrow). These images are representative of what was observed for all the cultures

To analyze the osteoclastic characteristics of the granuloma MGC and determine if the cells are hypersensitive to RANK-L and M-CSF, as demonstrated in the cherubism mouse model [14], we enzymatically digested the granulomas, cultured the cells in standard and osteoclastogenic media, and compared their characteristics with those of osteoclasts obtained from healthy-donor PBMC-ifferentiation cultures (control) (Figs. 4 and 5).

Five of the seven cases (1-B1, 1-B2, 1-C1, 1-C2, and 2B) yielded material suitable for granuloma cell culture. Cases 1-B2 and 1-C2 were only cultured in osteoclastic medium, and analyzed at day 7. In culture, TRAP-positive MGC with greater than 3 nuclei were considered as osteoclasts (Fig. 4). At day 14, the number of control osteoclasts/HPF varied from 0 to 6 (mean 3.6 ± 0.38), the number of nuclei per osteoclast varied from 3 to 6 (mean 3.2 ± 0.26), and the cell size varied from 49 μm to 122 μm (mean 88.2 μm ± 32) (Fig. 4).

Interestingly, in standard medium (i.e., without osteoclastogenic cytokines), the granuloma osteoclasts (day 7) were comparable in terms of both number/HPF (*p* = 0.053) and size (*p* = 0.09) to those in the control osteoclast culture (day 14, in osteoclastic medium), but showed a greater number of nuclei (*p* = 0.002) versus the control osteoclast culture (day 14, in osteoclastic medium) (Figs. 4 and 5). In addition, from day 3 to day 7, granuloma osteoclast number/HPF, number of nuclei per osteoclast, and osteoclast size increased (Additional file 9). This suggests that the cells from the cherubism granuloma are able to differentiate into osteoclasts even without M-CSF and RANKL supplementation to the culture medium.

In osteoclastic medium, granuloma osteoclasts (day 7) were comparable in terms of number/HPF (*p* = 0.75) compared to the control osteoclast culture (day 14), but they were larger (*p* = 0.02) and tended to have more nuclei (*p* = 0.03). In addition, from day 3 to day 7, granuloma osteoclast number/HPF, number of nuclei per osteoclast, and osteoclast size increased (Additional file 9). This suggests that cells of cherubism granulomas have a greater ability to fuse (Figs. 4 and 5).

Finally, in standard and osteoclastogenic media, granuloma cell cultures on bone slides generated resorption pits (Fig. 5), which seemed to be more prevalent when the cells were cultured in osteoclastogenic medium.

The results of the granuloma cell culture studies showed that the MGC could be considered as fully functional osteoclasts, as they are multinucleated (> 3 nuclei), TRAP-positive, and able to resorb bone. Granuloma cells can differentiate into osteoclasts and maturate without RANK-L and M-CSF medium supplementation (as evidenced by the increase in osteoclast number and size, and the number of nuclei between day 3 and day 7). However, these cells are still sensitive to RANK-L and M-CSF, because in osteoclastogenic medium the granuloma cells differentiate further into osteoclasts and the osteoclasts maturate from day 3 to day 7, and they generate more pits than when cultured in standard medium.

It must be noted that these cell cultures were not pure MGC cultures. Thus, the results of standard medium cultures suggest that granuloma cells might secrete cytokines which stimulate osteoclast differentiation, maturation and fusion. In addition, we cannot rule out that some MGC might have been present at the start of the culture.

### Cytokine analysis in culture supernatant

**Fig. 6:**
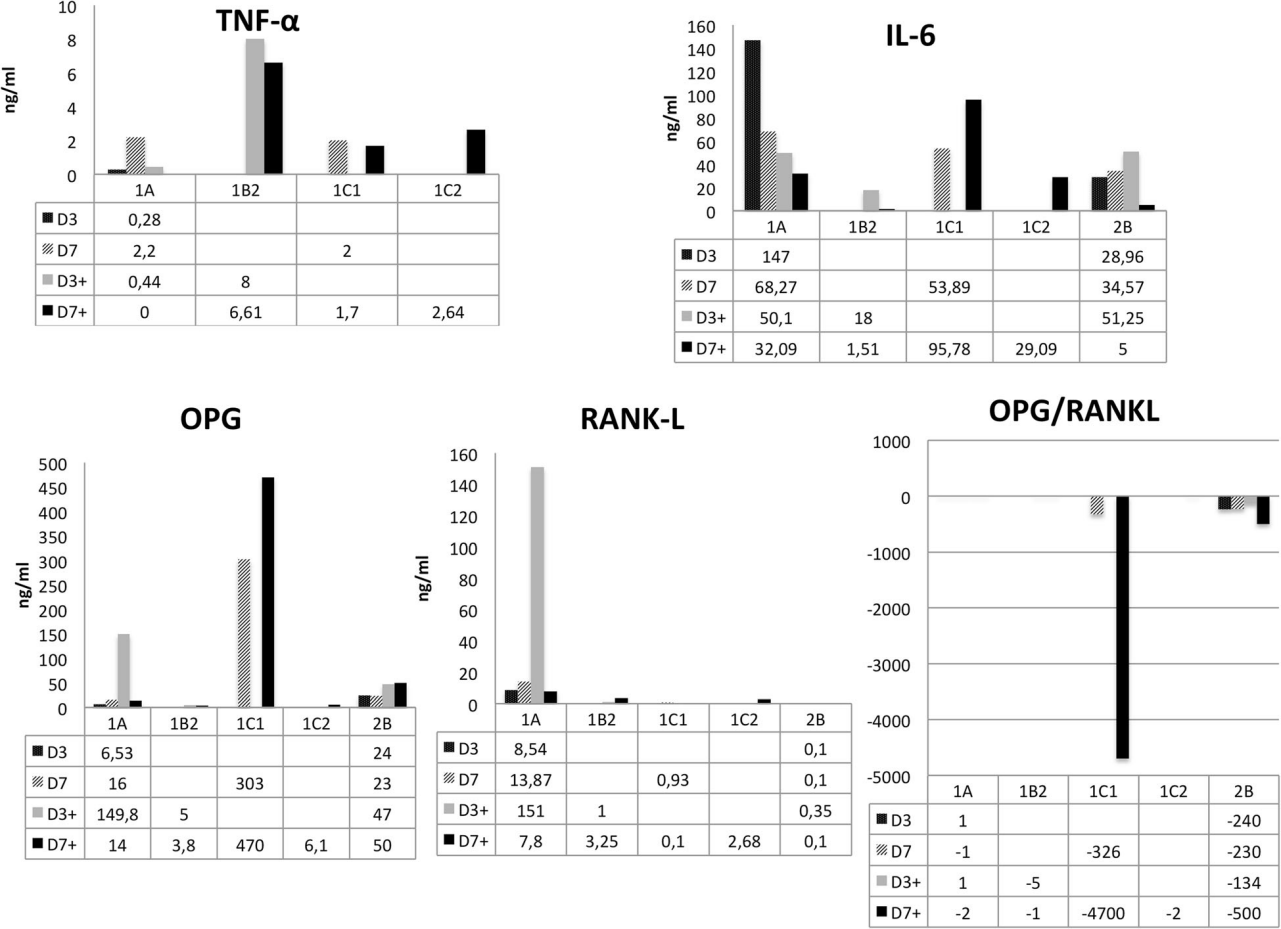
Cytokine expression in culture supernatant. The supernatant of both cell-culture types (standard medium and osteoclastogenic medium) was studied at day 3 and day 7. TNF-α was detected in the supernatant of all cultures. IL-6 was detected in the supernatant of all cultures at a higher level than TNF-α. OPG was detected in the supernatant of all cultures, whereas RANK-L levels were lower than OPG. D3 and D7 (3rd and 7th days of culture in standard medium), D3+ and D7+ (3rd and 7th days of culture in osteoclastogenic medium). RANK-L (receptor of activated nuclear factor kappa B ligand), OPG (osteoprotegerin), M-CSF (macrophage colony stimulating factor), IL (interleukin), TNF (tumor necrosis factor)

The main cytokines involved in auto-inflammatory bone diseases [16] are TNF-α, IL-1β, IL-17 and IL-6. Therefore, we next examined the levels of these cytokines in the culture supernatants by using ELISA (Fig. 6).

TNF-α was detected in the supernatant of all cultures (from 0 to 8 ng/ml, depending on the patient and culture conditions). IL-6 was detected in the supernatant of all cultures at higher levels than for TNF-α (from approx. 2 to 147 ng/ml, depending on the patient and culture conditions). IL-17 was not detected. OPG was detected in the supernatant of all cultures (from 5 to almost 150 ng/ml), whereas unbound RANK-L levels were generally lower than OPG levels (from barely detectable to 151 ng/ml), leading to a RANK-L/OPG ratio of less than 1 in most cases (Fig. 6).

Although in the culture supernatant the RANK-L/OPG ratio disfavors osteoclastogenesis, we demonstrated an increase in the number and size of the MGC in culture. The cytokine analysis showed that OPG and RANK-L are not playing their usual role in the osteoclastic differentiation of these granuloma cells, because even with the apparently unfavorable RANK-L/OPG ratio (< 1), the number of osteoclasts increased. Instead, IL-6 might stimulate osteoclast differentiation and maturation.

## Discussion

Cherubism is a rare disease described as an auto-inflammatory disease from the mouse model point of view. However human cherubism, and specially cherubism intraosseous lesion is poorly described in the literature. In the present study, we extensively (in situ and in vitro) explored cherubism intra-osseous lesions in order to determine if human cherubism could be considered as an auto-inflammatory bone disease. For this purpose, we explored cells in presence, and characterized the osteoclastic characteristics of MGC. Moreover, we examine the expression of inflammatory cytokines by granuloma cell cultures. Thus, we showed that human cherubism granuloma is composed mainly of osteoclasts or macrophages and relatively few immune cells, mainly CD68-positive cells, within a fibroblastic environment. The characteristics of these CD68-positive cells (macrophage vs. osteoclast) may predict the aggressiveness of the disease. Moreover, we demonstrated that the mechanism underlying human cherubism is different from that of mice; since, contrary to mice, it does not seem that TNF-α underlies the physiological mechanism of human cherubism, and that RANK-RANKL-OPG triad is not playing their usual role.

This study is the first large cellular and molecular analysis carried out on human cherubism granuloma samples. Our results show that MGC are derived from a monocyte lineage within a fibrous stroma with few inflammatory cells. The myeloid cells present in cherubism granuloma are mainly macrophages and osteoclasts, and the osteoclast phenotype may determine the aggressiveness of the disease. In addition, this study demonstrated that these osteoclast-like MGC are truly functional osteoclasts, able to resorb bone. Moreover, we showed a low expression of TNF-α pathway protein both in granuloma and in culture, while IL-6 is more widely expressed. In addition, it seems that the RANK/RANKL/OPG triad is disturbed in cherubism.

In this study, the seven unrelated patients presented an array of phenotypes, including both adult and childhood cases and non-aggressive to highly aggressive granulomas. Moreover, the study included a wide variety of analyses not previously performed in any study of human cherubism: ex vivo and in vitro studies, and pathological, immunohistological, biomolecular and cellular analyses.

The immunohistochemical analysis permitted us to characterize the types of cells present within the cherubism granuloma. Fibroblast cells and monocyte lineage cells are the most prevalent cells, with some rare lymphoid cells, principally CD8+ cells. These results support the definition of cherubism as an auto-inflammatory disease [17]. Auto-inflammatory bone disease is characterized by a chronic noninfectious inflammation which induces bone resorption, and results from aberrant activation of the innate immune system [16, 17]. Lymphoid cells and acquired molecules of the adaptive immune system do not play a role in this branch of auto-inflammatory bone diseases [26]. In cherubism mice, Ueki et al. previously demonstrated that cherubism is a myeloid (lymphoid independent) inflammatory bone-resorbing disease [14]. In our series, we detected four different cell types of myeloid cells: immature macrophages (CD68^+^, TRAP^−^ mononucleated cells), multinucleated macrophages (CD68^+^, TRAP^−^, nuclear NFATc1^−^ multinucleated cells), osteoclast precursors (CD68^+^, TRAP^+^ mononucleated cells), and osteoclasts (CD68^+^, TRAP^+^, nuclear NFATc1^+^ multinucleated cells). First, these results confirmed that MGC are derived from a monocyte lineage, as previously described [21]. In this study, the myeloid cell types present in granulomas are likely to predict the disease aggressiveness: i.e., osteoclast fate in aggressive cherubism and macrophage fate in non-aggressive cherubism, as we previously suggested [19]. Moreover, our in vitro analysis showed that the osteoclasts are functional and able to resorb bone, as was previously demonstrated by Southgate et al. [22].

Here, we discovered that RANKL has an unexpected expression in cherubism. We showed that the cherubism granulomas contained very few RANKL-positive cells (by immunohistochemistry), and these RANKL-positive cells were localized in regions poor in MGC. Moreover, *RANKL* mRNA expression was low in the granulomas; and in cultured cells, RANKL protein expression was also low. Furthermore, it seems that a negative feedback process is activated, with increased secretion of OPG. By immunohistochemistry, we showed numerous OPG-positive cells around MGC, in aggressive cherubism (1C1, 1C2). Liu et al. [21] showed similar results with extensive expression of OPG (by in situ hybridization). However, these results contrast with observations in mice. In *Sh3bp2* KI models, the observed osteopenia is due to an osteoblast functional defect and a reduction in OPG synthesis [27, 28]. In human cherubism, we hypothesize that osteoclast/MGC differentiation is induced through a RANK-L-independent pathway (because of a potential perpetual RANK activation), with inefficient regulation by OPG or a RANK-L bypass.

In the present study, one adult patient (2-A) in the remodeling phase was included. Interestingly, the patient’s granuloma displayed characteristics of osteogenesis: a RANK-L/OPG ratio less than 1, and high expression of ALP and OC (osteoblast markers).

Interestingly, cherubism cells differentiate into functional osteoclasts upon exogenous stimulation by RANK-L (Fig. 5c, d), but are also capable of differentiating without secreted or recombinant RANK-L stimulation (Fig. 5a, b) (Additional file 7). As suggested by Ueki et al. [14], mutated myeloid cells are hypersensitive to RANK-L and differentiate into osteoclasts, but may also differentiate through a RANK-L-independent pathway. Supporting these results, Mukai et al. [29] showed that *Sh3bp2*^*KI/+*^ bone marrow-derived M-CSF-dependent macrophages are highly sensitive to TNF-α and can differentiate into osteoclasts independently of RANK-L. Moreover, Wang et al. [30] suggested that cherubism bone phenotype may arise from a direct cross talk between osteoclast and osteoblast. Many mechanisms may explain the development of cherubism: other cytokines may be involved and may favor osteoclastogenesis in human cherubism independently of RANK-L; the RANK pathway may be permanently stimulated; or MGC may be stimulated through an autocrine pathway. All these theories may explain a supposed more accessory role of TNF-α in human cherubism.

These results suggest that cherubism cells produce a cytokine(s) that stimulates osteoclast differentiation (reflected in the increased number of osteoclasts from day 3 to day 7) and fusion (increased osteoclast size and nuclei from day 3 to day 7) in standard medium. Studies of mouse models of cherubism [14, 29, 31] suggest that TNF-α plays a key role in the disorder by enhancing osteoclast and macrophage differentiation. In humans, previously published immunohistochemical analyses showed TNF-α expression by MGC [29, 32]. In our study, immunohistochemical analysis did not show expression of TNF-R1 or R2 proteins, and biomolecular analysis showed *TNFR1*, *TNFR2* and *TNF-α* expression comparable to that in normal alveolar bone. Moreover, in vitro, the level of TNF-α in the culture supernatant was low. This result is in line with the dubious role of TNF-α in human cherubism. Indeed, Amaral et al. [33] showed that the relative expression of *TNF-α* transcript was low in cherubism, and others reported that anti-TNF-α therapy failed to improve cherubism lesions [32, 34]. Thus, osteoclast and macrophage differentiation must be stimulated by other cytokines. From our analysis of culture supernatant, IL-6 may be a likely candidate. IL-6 is widely recognized as a stimulator of bone loss in the context of inflammation, through pathways both dependent and independent of RANK-L [35–37].

This study is a first step to understand the molecular pathways leading to human cherubism. Our results suggest that osteoclast activation is mediated by translocation of NFATc1 into the nucleus, leading to the transcription of osteoclastogenic effectors such as TRAP. The role of NFATc1 had already been identified in the mouse model. However, in the *Sh3bp2* KI models, NFATc1 is implicated in bone loss but not in inflammation [15]. In human cherubism, three previous studies demonstrated the involvement of NFATc1 [4, 33, 38], showing an increase in NFATc1 transcription in cherubism granuloma and nuclear expression of NFATc1 in MGC. Moreover, we recently showed that nuclear NFATc1 expression is correlated with the prognosis for disease aggressiveness [19]. In the present study, we further described the osteoclast phenotype of MGC, the increased size of MGC (due to osteoclast fusion), and their self-maintenance. All these characteristics may be explained by the activation of NFATc1. Indeed, NFATc1 promotes its own amplification, the expression of osteoclastogenesis genes (such as TRAP), and osteoclast fusion [39].

Many challenges remain. First, because of the rarity of cherubism, a large prospective study is difficult to undertake. Second, the granulomas are highly variable not only between patients, but also within each granuloma itself. Thus, it is difficult to draw firm conclusions based on a single biopsy, and serial coring may be necessary. Finally, since MGC are osteoclasts (terminally differentiated cells), secondary cultures are impossible; indeed, serial analyses in cherubism cultures are difficult. Addressing these challenges will require investigation of a much larger series of patients, probably international in scope owing to the rarity of the disease, and creation of a human in vitro model.

## Conclusion

In summary, we showed that MGC are CD68-positive cells. These cells may differentiate into macrophages and osteoclasts, and their differentiation profile determines the aggressiveness of the disease. However, we do not know what triggers the choice between the osteoclast and macrophage lineage fates and hence the cherubism severity. In addition, we demonstrated that MGC differentiate through NFATc1-RANK-L-dependent and RANK-L-independent pathways whose activation is more likely due to IL-6 than to TNF-α. Our study brings new insights in the cellular mechanisms of human cherubism but leaves some important questions unresolved, such as the exclusive jaw bone localization, the absence of systemic disorders, and the natural history of the disease with its usually spontaneous resolution in adulthood.

## Additional files

Additional file 1: Primary antibodies used for automated immunohistochemistry. AE1-AE3 (pan cytokeratin), CD (cluster differentiation). (DOCX 14 kb)

Additional file 2: Primary, control and secondary antibodies for manual immunohistochemistry. NFATc1 (nuclear factor of activated T cells cytoplasmic 1), RANK-L (receptor of activated nuclear factor kappa B ligand), OPG (osteoprotegerin), RANK (receptor of activated nuclear factor kappa B), IL (interleukin), TNF-R1 (Tumor necrosis factor receptor 1). (DOCX 15 kb)

Additional file 3: List of the primers used for qPCR analysis. GAPDH (Glyceraldehyde 3-phosphate dehydrogenase), HPRT (hypoxanthine-guanine phosphoribosyltransferase), SDHA (succinate dehydrogenase complex subunit A), TBP (TATA-binding protein), RANK-L (receptor of activated nuclear factor kappa B ligand), OPG (osteoprotegerin), M-CSF (macrophage colony stimulating factor), NFATc1 (nuclear factor of activated T cells 1), RANK (receptor of activated nuclear factor kappa B), IL6-R (interleukin 6 receptor), IL6 (interleukin 6), TNF-R (tumor necrosis factor receptor), TNF-α (tumor necrosis factor α), ALP (alkaline phosphatase) (DOCX 16 kb)

Additional file 4: ELISA cytokine detection kit characteristics. RANK-L (receptor of activated nuclear factor kappa B ligand), OPG (osteoprotegerin), M-CSF (macrophage colony stimulating factor), IL (interleukin), TNF (tumor necrosis factor). (DOCX 15 kb)

Additional file 5: Biomolecular characteristics of cherubism granulomas. Results show the relative expression levels of RANKL, OPG, RANK, M-CSF, RANKL/OPG ratio, NFATc1, TNF-α, TNFr1, TNFr2, alkaline phosphatase (ALP), osteocalcin and OPG mRNA obtained by RT-qPCR on the surgical specimens. Tumors and bone expressed M-CSF, TNF-α, TNF-R1, TNF-R2 mRNA without significant differences. RANK mRNA was more expressed in cases 1-B2 and 1-C1 (*p* = 0.003). RANK-L mRNA was significantly more expressed in 1-B2 (*p* = 0.012). OPG mRNA was significantly more expressed in 2-A (*p* = 0.0002). RANKL/OPG ratio was positive in all cases but 2-A. NFATc1 mRNA was significantly increased in 1-B2 (*p* = 0.0001). Osteocalcin and ALP mRNA were significantly more expressed in 2-A (*p* < 0.0001). (TIF 26330 kb)

Additional file 6: Bone remodeling marker: RANK-L, OPG, RANK, NFATc1 immunohistochemistry (control with secondary antibody). A: Case 1-C2; RANK control (scale bar = 200 μm). B: Case 1-C1: OPG control (scale bar = 200 μm). C: Case 1-C1: RANKL control (scale bar = 200 μm). D: Case 1-C2:NFATc1 control (scale bar = 100 μm). (TIF 26330 kb)

Additional file 7: Patient 1B2 HES and Immunohistochemistry photographs. (AE1/AE3, CD3, CD5, CD20, CD68, CD8, CD4, Vimentine, NFATc1, OPG, RANK scale bar = 50 μm; TRAP activity and RANK scale bar = 100 μm, HES scale bar = 200 μm). Granuloma did not expressed AE1/AE2. Few stromal cells expressed CD3 and CD5. No cells expressed CD20. Few stromal cells expressed CD4 and CD8. Stromal cells and MGC expressed CD68. All cells expressed vimentine. Granuloma cells did not expressed RANK, OPG and NFATc1. Some stromal cells expressed RANKL. TRAP assay were negative. In HES, the granuloma showed collagen-rich fibrous area with few MGC. (TIF 18313 kb)

Additional file 8: Patient 1C1 HES and Immunohistochemistry photographs. (AE1/AE3, CD3, CD5, CD20, CD68, CD8, CD4, Vimentine, NFATc1, OPG, RANK scale bar = 50 μm; TRAP activity and RANK scale bar = 100 μm, HES scale bar = 200 μm). Granuloma did not expressed AE1/AE2. Some stromal cells expressed CD3 and CD5. Few cells expressed CD20 and CD4. Some stromal cells expressed CD8. Stromal cells and MGC expressed CD68. All cells expressed vimentine. Stromal and MGC cells expressed RANK. Some stromal cells expressed RANKL and OPG. TRAP assay were positive for MGC. NFATc1 was expressed in the nuclei and cytoplasm of MCG. In HES, the granuloma showed intralesional hemorrhage with many MCG. (TIF 18313 kb)

Additional file 9: Cherubism giant multinucleated cells (MGC) A-C: Standard medium cultures: MGC differentiate into osteoclasts (TRAP-positive cells, > 3 nuclei). A. Osteoclast number increased from day 3 to day 7. B. Nuclei number per osteoclast increased from day 3 to day7. C: size of osteoclasts increased from day 3 to day 7. D-F: Osteoclastogenic medium cultures: MGC are sensitive to RANKL and M-CSF. D: Osteoclast number increased from day 3 to day 7 in culture with osteoclastogenic medium. E: Nuclei number per osteoclast increased from day 3 to day 7 in culture with osteoclastogenic medium. F. Size of osteoclasts increased from day 3 to day 7 in culture with osteoclastogenic medium. RANK-L (receptor of activated nuclear factor kappa B ligand), M-CSF (macrophage colony stimulating factor), TRAP: tartrate resistant acid phosphatase (TIF 1304 kb)

## Abbreviations

AE1-AE3: Multicytokeratin
ALP: Alkaline phosphatase
CD: Cluster differentiation
DAB: Diaminobenzidine
ELISA: Enzyme linked immunosorbent assay
GAPDH: Glyceraldehyde 3-phosphate dehydrogenase
HES: Hematoxylin - eosin - safranin
HPF: High-power field
HPRT: Hypoxanthine-guanine phosphoribotransferase
IL: Interleukin
IL6-R: Interleukin-6 receptor
M-CSF: Macrophage colony-stimulating factor
MGC: Multinucleated giant cell
NFAT: Nuclear factor of activated T-cells
OPG: Osteoprotegerin
PBMC: Peripheral blood mononuclear cell
PBS: Phosphate-buffered saline
RANK: Receptor activator of nuclear factor kappa-B
RANK-L: Receptor activator of nuclear factor kappa-B ligand
RNA: Ribonucleic acid
RTqPCR: Reverse transcription quantitative polymerase chain reaction
SDHA: Succinate dehydrogenase complex subunit A
SH3BP2: SH3 domain-binding protein 2
Syk: Spleen tyrosine kinase
TBP: TATA-binding protein
TNF: Tumor necrosis factor
TNF-R: Tumor necrosis factor receptor
TRAP: Tartrate resistant acid phosphatase
