# Supplementary material for: Molecular and cellular characterizations of human cherubism: disease aggressiveness depends on osteoclast differentiation
Source: Orphanet J Rare Dis. 2018 Sep 20;13:166. doi: 10.1186/s13023-018-0907-2 (PMC6148781; doi:10.1186/s13023-018-0907-2)
Supplement: Supplementary file 1 — Primary antibodies used for automated immunohistochemistry. AE1-AE3 (pan cytokeratin), CD (cluster differentiation). (DOCX 14 kb) [file 13023_2018_907_MOESM1_ESM.docx]

**Additional file 1: Primary antibodies used for automated immunohistochemistry.**

AE1-AE3 (pan cytokeratin), CD (cluster differentiation)

| **Primary Antibody** | **Manufacturer** | **Reference** | **Isotype** | **Species** | **pH for antigen retrieval** | **Dilution** |
| --- | --- | --- | --- | --- | --- | --- |
| **AE1-AE3** | DAKO | M3515 | IgG1 | Mouse | 6 | 1:50 |
| **CD68** | DAKO | M0814 | IgG1 | Mouse | 6 | 1:500 |
| **CD4** | DAKO | M7310 | IgG1 | Mouse | 6 | 1:20 |
| **CD8** | DAKO | M7103 | IgG1 | Mouse | 6 | 1:25 |
| **CD3** | MM-France | F/RM9107-S1 | IgG1 | Mouse | 6 | 1:100 |
| **CD5** | LEICA | CD5-4C7-L-CE | IgG1 | Mouse | 6 | 1:30 |
| **CD20** | DAKO | M0755 | IgG2a | Mouse | 6 | 1:100 |
| **Vimentin** | LEICA | VIM-V9-L-CE | IgG1 | Mouse | 6 | 1:50 |
