# Supplementary material for: Molecular and cellular characterizations of human cherubism: disease aggressiveness depends on osteoclast differentiation
Source: Orphanet J Rare Dis. 2018 Sep 20;13:166. doi: 10.1186/s13023-018-0907-2 (PMC6148781; doi:10.1186/s13023-018-0907-2)
Supplement: Supplementary file 2 — Primary, control and secondary antibodies for manual immunohistochemistry. NFATc1 (nuclear factor of activated T cells cytoplasmic 1), RANK-L (receptor of activated nuclear factor kappa B ligand), OPG (osteoprotegerin), RANK (receptor of activated nuclear factor kappa B), IL (interleukin), TNF-R1 (Tumor necrosis factor receptor 1). (DOCX 15 kb) [file 13023_2018_907_MOESM2_ESM.docx]

**Additional file 2: Primary, control and secondary antibodies for manual immunohistochemistry.** NFATc1 (nuclear factor of activated T cells cytoplasmic 1), RANK-L (receptor of activated nuclear factor kappa B ligand), OPG (osteoprotegerin), RANK (receptor of activated nuclear factor kappa B), IL (interleukin), TNF-R1 (Tumor necrosis factor receptor 1).

|  |  | **Manufacturer** | **Reference** | **Isotype** | **Species** | **pH for antigen retrieval** | **Concentration** |
| --- | --- | --- | --- | --- | --- | --- | --- |
| **Primary Antibody** | **NFAT-c1** | Santa-Cruz | SC-7294 | IgG1 | Mouse | 6 | 2μg/ml |
|  | **RANK-L** | R&D Systems | 70525 | IgG2 | Mouse | 6 | 2.5μg/ml |
|  | **OPG** | R&D Systems | 69146 | IgG1 | Mouse | 6 | 6.5μg/ml |
|  | **RANK** | Santa-Cruz | SC-7625 | Polyclonal | Goat | 9 | 5μg/ml |
|  | **TNF-R1** | Santa-Cruz | SC-7895 | Polyclonal | Rabbit | 6 | 5μg/ml |
|  | **IL-6** | Ab-Cam | Ab128008 | Polyclonal | Rabbit | 6 | 3μg/ml |
| **Control Antibody** | **Control IgG1 mouse** | Dako | X-0931 | IgG1 | Mouse | 6 | 100μg/ml |
|  | **Control IgG2 mouse** | Santa-Cruz | SC-2027 | IgG | Mouse | 6 | 100μg/ml |
|  | **Control goat** | Sigma | I-5256 | Polyclonal | Goat | 6 | 1000μg/ml |
|  | **Control rabbit** | Dako | X-0903 | Polyclonal | Rabbit | 6 | 20μg/ml |
| **Secondary antibody** | **Biotin anti goat IgG** | Jackson | 705-066-147 | NA | Donkey | 6 | 1700μg/ml |
|  | **Biotin anti mouse IgG** | Jackson | 715-066-150 | NA | Donkey | 6 | 1100μg/ml |
|  | **Biotin anti rabbit IgG** | Jackson | 711-066-152 | NA | Donkey | 6 | 1200μg/ml |
