# Supplementary material for: Molecular and cellular characterizations of human cherubism: disease aggressiveness depends on osteoclast differentiation
Source: Orphanet J Rare Dis. 2018 Sep 20;13:166. doi: 10.1186/s13023-018-0907-2 (PMC6148781; doi:10.1186/s13023-018-0907-2)
Supplement: Supplementary file 3 — List of the primers used for qPCR analysis. GAPDH (Glyceraldehyde 3-phosphate dehydrogenase), HPRT (hypoxanthine-guanine phosphoribosyltransferase), SDHA (succinate dehydrogenase complex subunit A), TBP (TATA-binding protein), RANK-L (receptor of activated nuclear factor kappa B ligand), OPG (osteoprotegerin), M-CSF (macrophage colony stimulating factor), NFATc1 (nuclear factor of activated T cells 1), RANK (receptor of activated nuclear factor kappa B), IL6-R (interleukin 6 receptor), IL6 (interleukin 6), TNF-R (tumor necrosis factor receptor), TNF-α (tumor necrosis factor α), ALP (alkaline phosphatase) (DOCX 16 kb) [file 13023_2018_907_MOESM3_ESM.docx]

**Additional file 3: List of the primers used for qPCR analysis.** GAPDH (Glyceraldehyde 3-phosphate dehydrogenase), HPRT (hypoxanthine-guanine phosphoribosyltransferase), SDHA (succinate dehydrogenase complex subunit A), TBP (TATA-binding protein), RANK-L (receptor of activated nuclear factor kappa B ligand), OPG (osteoprotegerin), M-CSF (macrophage colony stimulating factor), NFATc1 (nuclear factor of activated T cells 1), RANK (receptor of activated nuclear factor kappa B), IL6-R (interleukin 6 receptor), IL6 (interleukin 6), TNF-R (tumor necrosis factor receptor), TNF-α (tumor necrosis factor α), ALP (alkaline phosphatase)

| **Primers** | **Forward 5’-3’** | **Reverse 5’-3’** |
| --- | --- | --- |
| ***GAPDH*** | CCT-GGA-GGC-TAT-CCA-GCG-TA | GGA-GAC-GCC-AGT-GGA-CTC-CA |
| ***HPRT*** | TGA-CAC-TGG-CAA-AAC-AAT-GCA | GGT-CCT-TTT-CAC-CAG-CAA-GCT |
| ***SDHA*** | TGG-GAA-CAA-GAG-GGC-ATC-TG | CCA-CCA-CTG-CAT-CAA-AAT-TCA-TG |
| ***TBP*** | CCC-GAA-ACG-CCG-AAT-ATA-ATC-C | GAC-TGT-TCT-TCA-CTC-TTG-GCT-C |
| ***RANKL*** | TGA-TTC-ATG-TAG-GAG-AAT-TAA-ACA-GG | GAT-GTG-CTG-TGA-TCC-AAC-GA |
| ***OPG*** | GCG-CTC-GTG-TTT-CTG-GAC-A | AGT-ATA-GAC-ACT-CGT-CAC-TGG-TG |
| ***M-CSF*** | GTG-CCC-AGC-AGC-AGC-TGG-AA | GGC-CAG-GAT-GGG-CAG-AGA-TGG-GG |
| ***RANK*** | AGA-TCG-CTC-CTC-CAT-GTA-CCA | GCC-TTG-CCT-GTA-TCA-CAA-ACT-T |
| ***NFATc1*** | GCC-CCA-GAT-GGC-CAC-CAT-GTC-T | AGC-ACC-CCA-CGC-GCT-CAT-GT |
| ***IL6*** | CCA-GAG-CTG-TGC-AGA-TGA | CTG-CAG-CCA-CTG-GGT-CTG-T |
| ***IL6-R*** | GTA-CCA-CTG-CCC-ACA-TTC-CT | CAG-CTT-CCA-CGT-CTT-CTT-GA |
| ***TNF-R1*** | CTC-TCC-ACC-GTG-CCT-GAC | CCA-GTC-CAA-TAA-CCC-CTG-AG |
| ***TNF-R2*** | CAG-TGC-GTT-GGA-CAG-AAG-G | CCA-CCA-GGG-GAA-GAA-TCT-G |
| ***TNF-α*** | CGT-GGC-TAA-GAA-TGT-CAT-CAT-GTT | TGG-AGC-TGA-CCC-TTG-AGG-AT |
| ***ALP*** | CGT-GGC-TAA-GAA-TGT-CAT-CAT-GTT | TGG-AGC-TGA-CCC-TTG-AGG-AT |
| ***Osteocalcine*** | GTG-CAG-AGT-CCA-GCA-AAG-GT | AAA-GAA-GGG-TGC-CTG-GAG |
