# Supplementary material for: Molecular and cellular characterizations of human cherubism: disease aggressiveness depends on osteoclast differentiation
Source: Orphanet J Rare Dis. 2018 Sep 20;13:166. doi: 10.1186/s13023-018-0907-2 (PMC6148781; doi:10.1186/s13023-018-0907-2)
Supplement: Supplementary file 4 — ELISA cytokine detection kit characteristics. RANK-L (receptor of activated nuclear factor kappa B ligand), OPG (osteoprotegerin), M-CSF (macrophage colony stimulating factor), IL (interleukin), TNF (tumor necrosis factor). (DOCX 15 kb) [file 13023_2018_907_MOESM4_ESM.docx]

**Additional file 4 : ELISA cytokine detection kit characteristics**

RANK-L (receptor of activated nuclear factor kappa B ligand), OPG (osteoprotegerin), M-CSF (macrophage colony stimulating factor), IL (interleukin), TNF (tumor necrosis factor)

| **Cytokine** | **Manufacturer** | **References** | **Lower detection limit (pg/ml)** | **Higher detection limit (pg/ml)** |
| --- | --- | --- | --- | --- |
| **RANK-L** | R&D Systems | DY626 | 78.1 | 5000 |
| **OPG** | R&D Systems | DY805 | 62.5 | 4000 |
| **M-CSF** | R&D Systems | DY216 | 15.6 | 1000 |
| **TNF-α** | R&D Systems | DY210 | 15.6 | 1000 |
| **IL-6** | R&D Systems | DY206 | 9.38 | 600 |
