# Supplementary figures and images for: Molecular and cellular characterizations of human cherubism: disease aggressiveness depends on osteoclast differentiation
Source: Orphanet J Rare Dis. 2018 Sep 20;13:166. doi: 10.1186/s13023-018-0907-2 (PMC6148781; doi:10.1186/s13023-018-0907-2)

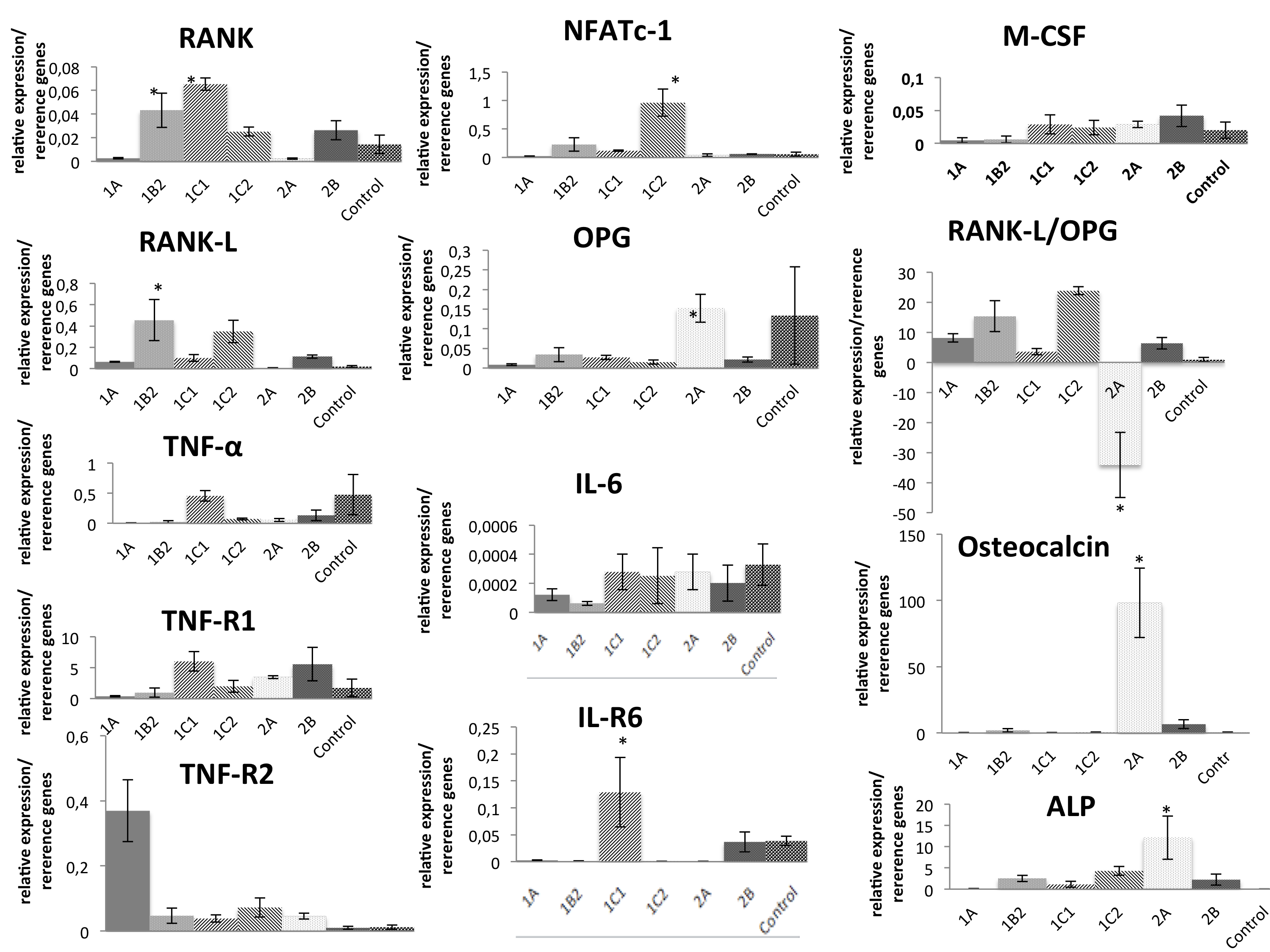

Supplement: Supplementary file 5 — Biomolecular characteristics of cherubism granulomas. Results show the relative expression levels of RANKL, OPG, RANK, M-CSF, RANKL/OPG ratio, NFATc1, TNF-α, TNFr1, TNFr2, alkaline phosphatase (ALP), osteocalcin and OPG mRNA obtained by RT-qPCR on the surgical specimens. Tumors and bone expressed M-CSF, TNF-α, TNF-R1, TNF-R2 mRNA without significant differences. RANK mRNA was more expressed in cases 1-B2 and 1-C1 (p = 0.003). RANK-L mRNA was significantly more expressed in 1-B2 (p = 0.012). OPG mRNA was significantly more expressed in 2-A (p = 0.0002). RANKL/OPG ratio was positive in all cases but 2-A. NFATc1 mRNA was significantly increased in 1-B2 (p = 0.0001). Osteocalcin and ALP mRNA were significantly more expressed in 2-A (p < 0.0001). (TIF 26330 kb) [file 13023_2018_907_MOESM5_ESM.tif]

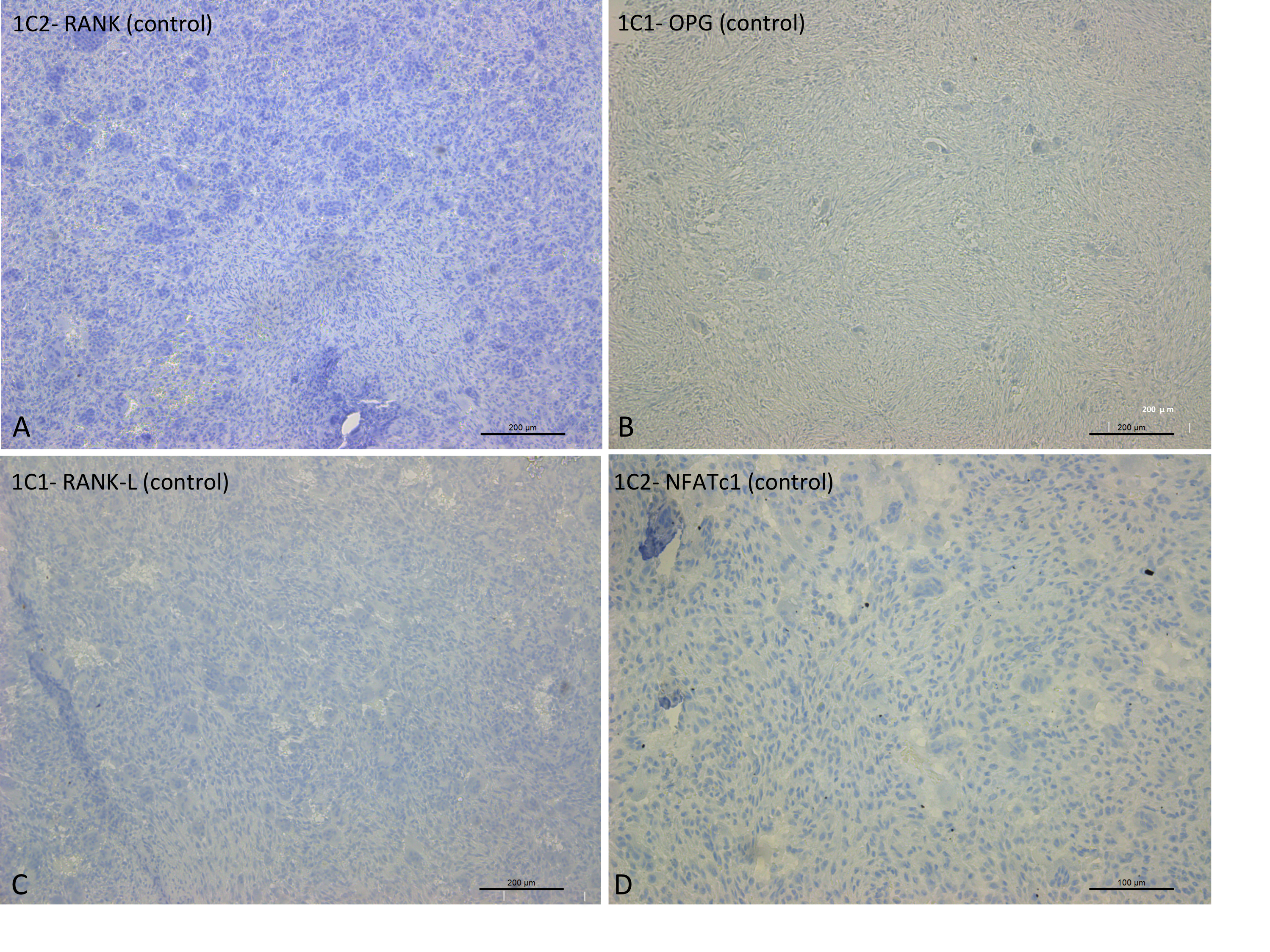

Supplement: Supplementary file 6 — Bone remodeling marker: RANK-L, OPG, RANK, NFATc1 immunohistochemistry (control with secondary antibody). A: Case 1-C2; RANK control (scale bar = 200 μm). B: Case 1-C1: OPG control (scale bar = 200 μm). C: Case 1-C1: RANKL control (scale bar = 200 μm). D: Case 1-C2:NFATc1 control (scale bar = 100 μm). (TIF 26330 kb) [file 13023_2018_907_MOESM6_ESM.tif]

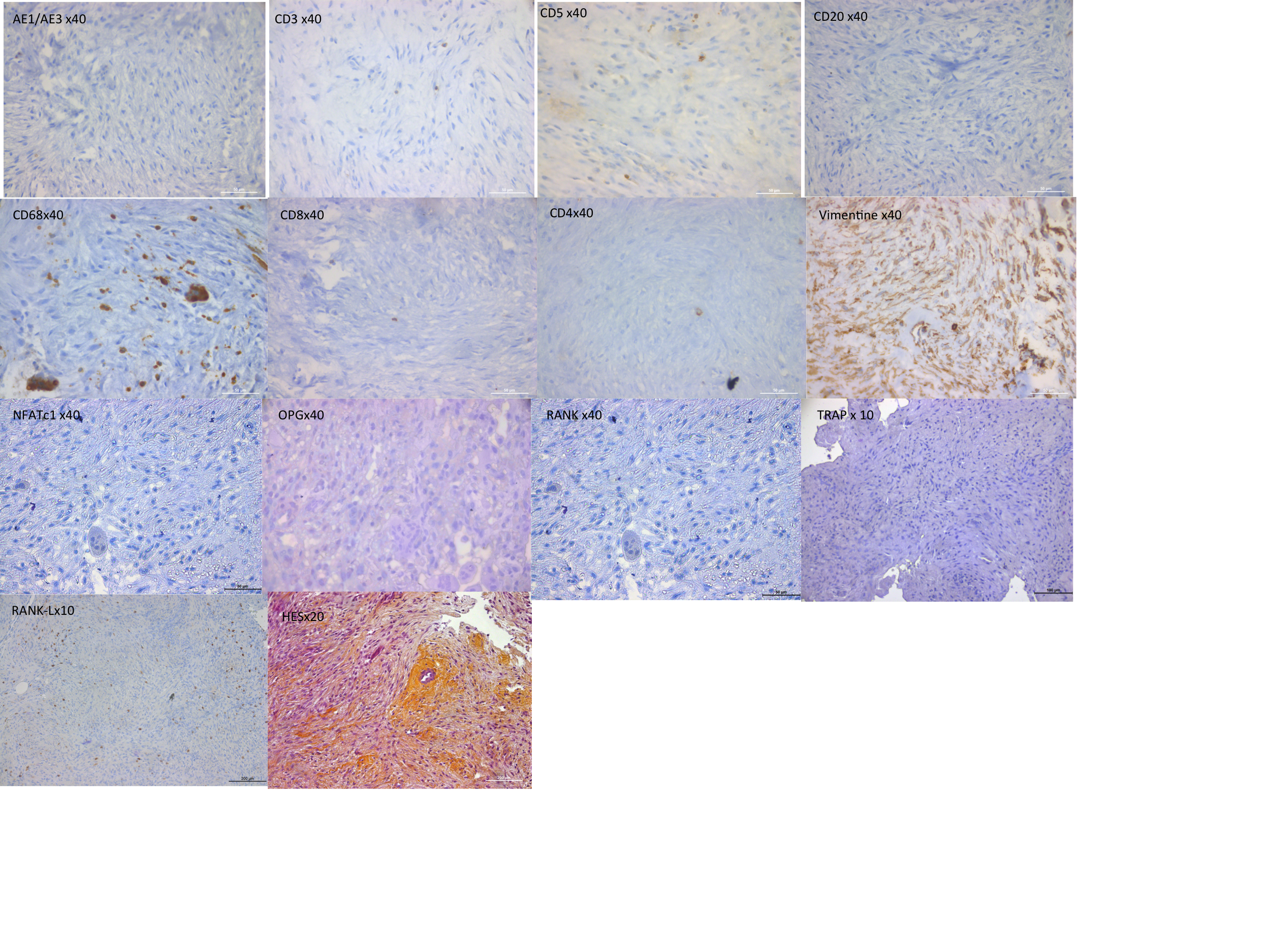

Supplement: Supplementary file 7 — Patient 1B2 HES and Immunohistochemistry photographs. (AE1/AE3, CD3, CD5, CD20, CD68, CD8, CD4, Vimentine, NFATc1, OPG, RANK scale bar = 50 μm; TRAP activity and RANK scale bar = 100 μm, HES scale bar = 200 μm). Granuloma did not expressed AE1/AE2. Few stromal cells expressed CD3 and CD5. No cells expressed CD20. Few stromal cells expressed CD4 and CD8. Stromal cells and MGC expressed CD68. All cells expressed vimentine. Granuloma cells did not expressed RANK, OPG and NFATc1. Some stromal cells expressed RANKL. TRAP assay were negative. In HES, the granuloma showed collagen-rich fibrous area with few MGC. (TIF 18313 kb) [file 13023_2018_907_MOESM7_ESM.tif]

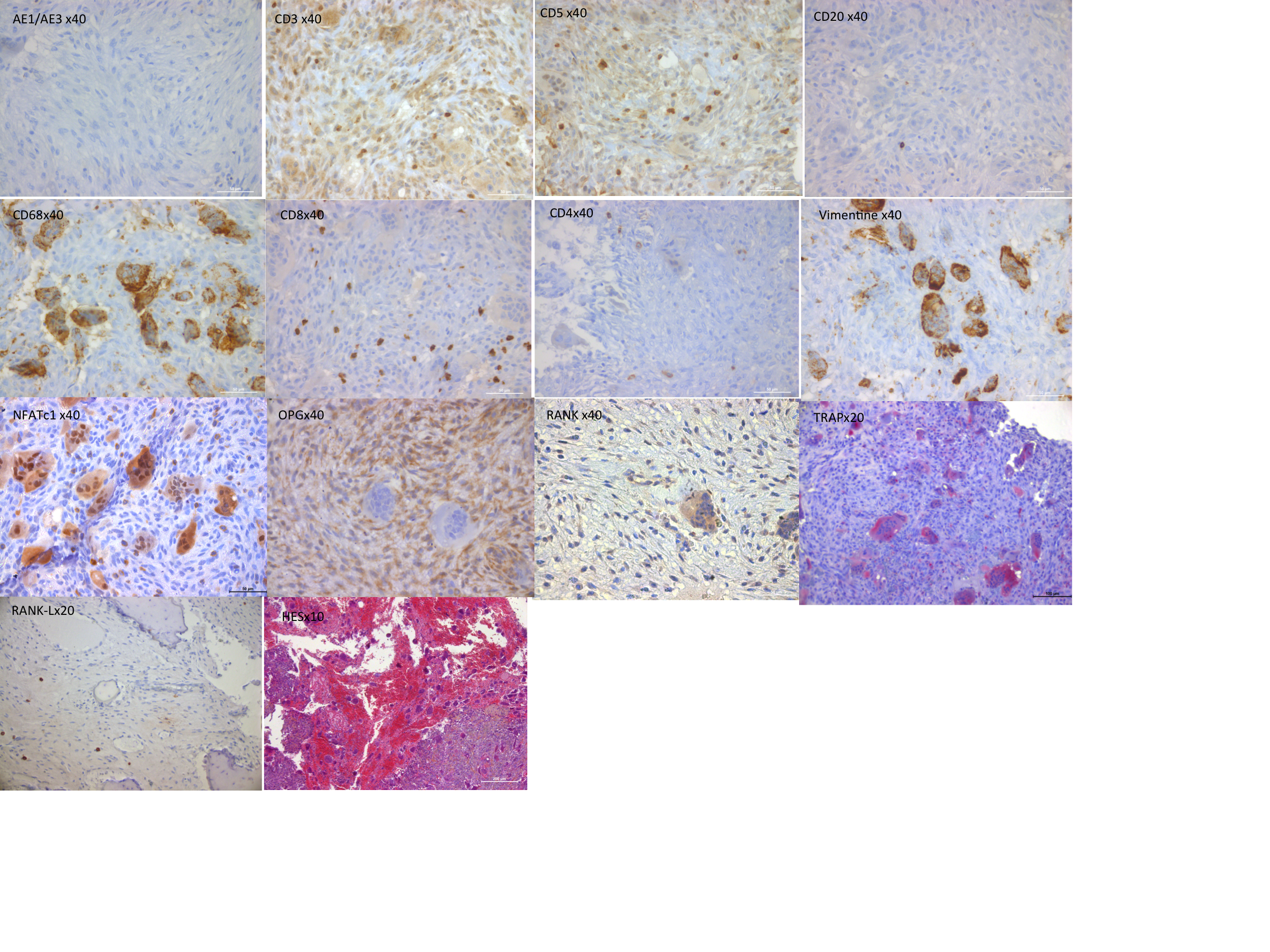

Supplement: Supplementary file 8 — Patient 1C1 HES and Immunohistochemistry photographs. (AE1/AE3, CD3, CD5, CD20, CD68, CD8, CD4, Vimentine, NFATc1, OPG, RANK scale bar = 50 μm; TRAP activity and RANK scale bar = 100 μm, HES scale bar = 200 μm). Granuloma did not expressed AE1/AE2. Some stromal cells expressed CD3 and CD5. Few cells expressed CD20 and CD4. Some stromal cells expressed CD8. Stromal cells and MGC expressed CD68. All cells expressed vimentine. Stromal and MGC cells expressed RANK. Some stromal cells expressed RANKL and OPG. TRAP assay were positive for MGC. NFATc1 was expressed in the nuclei and cytoplasm of MCG. In HES, the granuloma showed intralesional hemorrhage with many MCG. (TIF 18313 kb) [file 13023_2018_907_MOESM8_ESM.tif]

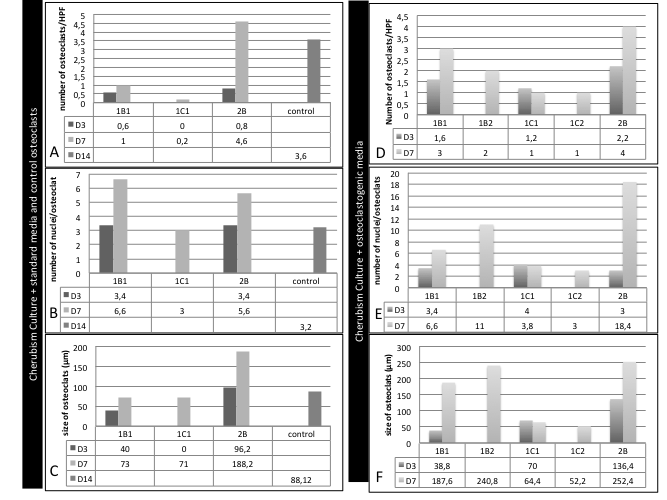

Supplement: Supplementary file 9 — Cherubism giant multinucleated cells (MGC) A-C: Standard medium cultures: MGC differentiate into osteoclasts (TRAP-positive cells, > 3 nuclei). A. Osteoclast number increased from day 3 to day 7. B. Nuclei number per osteoclast increased from day 3 to day7. C: size of osteoclasts increased from day 3 to day 7. D-F: Osteoclastogenic medium cultures: MGC are sensitive to RANKL and M-CSF. D: Osteoclast number increased from day 3 to day 7 in culture with osteoclastogenic medium. E: Nuclei number per osteoclast increased from day 3 to day 7 in culture with osteoclastogenic medium. F. Size of osteoclasts increased from day 3 to day 7 in culture with osteoclastogenic medium. RANK-L (receptor of activated nuclear factor kappa B ligand), M-CSF (macrophage colony stimulating factor), TRAP: tartrate resistant acid phosphatase (TIF 1304 kb) [file 13023_2018_907_MOESM9_ESM.tif]
